# A Multiomics and Bioinformatics Analysis of the Role and Mechanism of *CNKSR1* in Ovarian Cancer Progression

**DOI:** 10.1155/ijog/1546020

**Published:** 2026-04-11

**Authors:** Weili Zhu, Yimin Huang, Jiayue Huang, Jianguo Wang, Sijia Shen

**Affiliations:** ^1^ Jiaxing Maternity and Child Health Care Hospital, Jiaxing, Zhejiang, 314001, China, jxfby.com

**Keywords:** *CNKSR1*, DEGs, EMT, metastasis, OC, transcriptome sequencing

## Abstract

This study aims to utilize multiomics and bioinformatics techniques to investigate the expression of the connection enzyme inhibitor Ras1 1 (CNKSR1) in ovarian cancer (OC) and its mechanism of action in the processes of tumor proliferation, invasion, and metastasis. Transcriptome sequencing was performed on paired cancerous and normal ovarian tissues from three OC patients. Differentially expressed genes (DEGs) mRNAs were analyzed using “limma”, and key DEGs were identified through LASSO and random forest (RF) methods. *CNKSR1* expression in OC tissues was assessed via quantitative reverse transcription polymerase chain reaction (qRT‐PCR), Western blot, and immunohistochemistry. Gene Ontology (GO) enrichment analysis was conducted utilizing the Database for Annotation, Visualization, and Integrated Discovery (DAVID), and CancerSEA was employed to assess the functional status of *CNKSR1* in OC. Also, the proliferation of cells, their invasion, and migration were evaluated. ChEA3 and hTFtarget were used to identify potential transcription factors targeting *CNKSR1*. The predicted factors were validated by Western blot analysis in OC cells overexpressing *CNKSR1* or in OC cells with the *CNKSR1* knockdown. Compared to normal tissues, 78 genes were downregulated, and 101 were upregulated in OC. LASSO and RF analyses identified *CNKSR1* as a vital gene positively correlated with the progression of OC, which was then experimentally validated. *CNKSR1* upregulation promoted the OC cells’ proliferation, their invasion, and migration, while its silencing inhibited these processes. Analysis of the ChEA3 and hTFtarget databases, as well as Western blot analysis, suggested that *CNKSR1* may drive epithelial‐mesenchymal transition (EMT) by upregulating snail and ZEB1 expression. *CNKSR1* serves a significant role in promoting the growth and progression of OC. It enhances OC cells’ metastatic and invasive abilities by regulating the EMT mediated by the transcription factors Snail and ZEB1.

## 1. Introduction

Ovarian cancer (OC) is among the deadliest female reproductive system malignancies, with a worldwide prevalence rate of 1.6% and a mortality rate of 5.8% (Stewart, Ralyea and Lockwood, 2019; Sung et al., 2021). Despite significant advances in surgery, chemotherapy, and molecular‐targeted therapies over the past two decades, survival rates of patients with advanced OC remain low (Lheureux, Braunstein and Oza, 2019; Lee, Minasian and Kohn, 2019). Studies have shown that molecular‐targeted therapies, like Programmed Cell Death Protein 1 (PD‐1) inhibitors and Epidermal Growth Factor Receptor (EGFR)‐targeting drugs, are only effective in certain patients (Glaysher et al., 2013; Fan, Reader and Roque, 2018; Han, Liu and Li, 2020). Additionally, although molecular‐targeted drugs such as gefitinib, dasatinib, and erlotinib have shown improved clinical outcomes when used in combination with chemotherapeutic agents, they are associated with high costs and implementation difficulties (Thibault and Jean‐Claude, 2017; Li et al., 2023b). Therefore, identifying new biomarkers to enhance diagnostic accuracy and improve survival rates in OC patients has become a pressing public health issue.

CNKSR1 proteins, also known as Connector Enhancer of KSR1 (CNK1), are evolutionarily conserved scaffolds that regulate various signaling pathways (Fritz and Radziwill, 2011). Numerous studies have indicated that the *CNKSR1* gene and its encoded protein are significantly elevated in tumors such as lung adenocarcinoma and pancreatic cancer, where *CNKSR1* assumes a critical function in malignant cell signaling, multiplication, and invasion, thereby promoting tumor development (Fritz and Radziwill, 2010; Fritz, Varga and Radziwill, 2010; Quadri et al., 2017; Cai and Peng, 2024).

Research has shown that the knockdown of *CNKSR1* expression can inhibit proliferation in breast cancer and human embryonic kidney cells, while overexpression of *CNKSR1* stimulates proliferation in these cells (Fritz, Varga, and Radziwill, 2010). A study by Quadri et al. reported high *CNKSR1* expression in pancreatic cancer cells, which positively correlated with a poor prognosis (Quadri et al., 2017). Studies have also found that *CNKSR1* mRNA expression levels negatively correlate with expression of PD‐L1 mRNA expression and infiltration of immune cells in lung adenocarcinoma (Cai and Peng, 2024). Fritz et al. demonstrated that *CNKSR1* knockdown reduces the invasive capabilities of breast and cervical cancer cells (Fritz and Radziwill, 2010). A recent study also showed that *CNKSR1* served as a regulatory element in the adaptive resistance to MEK suppression, facilitating crosstalk with AKT signaling through its scaffolding role for the phosphorylated variant of AKT (Li et al., 2023a). While all these findings reveal the critical role of *CNKSR1* in signaling, invasion, and propagation of malignant cells, the expression level and regulatory mechanisms of *CNKSR1* in OC remain uncertain. To date, the expression status and functional relevance of *CNKSR1* in OC have not been elucidated, which motivated the present study.

The objective of this study is to (1) characterize *CNKSR1* expression in OC tissues and cell lines; (2) assess its effects on OC cell proliferation, migration, and invasion; and (3) dissect the molecular mechanism by which *CNKSR1* regulates OC metastasis, with a focus on the epithelial‐mesenchymal transition (EMT) pathway.

## 2. Materials and Methods

### 2.1. Cell Cultures and Reagents

The study’s reagents were sourced from Millipore Sigma situated in St. Louis, MO, USA, if not otherwise specified.

The human OC cell lines A2780, OVCAR3, SKOV3, and Hey, along with the human typical ovarian cell line IOSE80, were acquired from Cas9X located in Suzhou, Jiangsu, China. The cells were grown in Dulbecco’s Modified Eagle Medium (DMEM, Hyclone) supplemented with 10% fetal bovine serum (FBS, Gibco), 100 U/mL penicillin, and 100 μg/mL streptomycin, controlled at 37°C and 5% CO_2_.

### 2.2. Lentivirus Production and Transfection

The *CNKSR1* overexpression plasmid (plenti‐CNKSR1), empty vector (plenti‐Negative), *CNKSR1* small hairpin RNA (shRNA) plasmid (pLKO‐shCNKSR1), shRNA empty vector (shCNKSR1‐Negative), and lentiviral vectors were obtained from Sangon Biotech (Shanghai, China). *CNKSR1* overexpression in the OC cell line SKOV3 was achieved by lentiviral packaging and transfection. Initially, HEK293T cells were used for lentivirus packaging and co‐transfected with three plasmids: the target expression plasmid (pLKO‐shCNKSR1 or plenti‐CNKSR1), packaging plasmid (psPAX2), and envelope plasmid (pMD2.G) at a 4:3:1 ratio. The cells were transfected utilizing Lipofectamine 2000 (Thermo Fisher Scientific, Waltham, MA, USA). Following transfecting for 24 h, fresh antibiotic‐free medium was used as a substitute for the culture medium. The virus‐containing supernatant was harvested 48 and 72 h after the transfection, and a 0.45 µm membrane was used to filter it to eradicate cellular particles. The filtered viral supernatant was then used to infect target cells, which were typically at 30%–50% confluency at the time of infection. Addition of Polybrene (4 µg/mL) was performed to bolster infection efficacy. Following an incubation period of 12 to 24 h, fresh culture medium was introduced to replace the existing medium, and fluorescent microscopy was used to screen for positive cells 48 h postinfection. For negative control, a blank vector was employed; sh‐CNKSR1 was used to suppress *CNKSR1* expression, with an empty shRNA vector serving as the negative control. Preliminary dose‐escalation experiments confirmed that 1 mg/mL G418 effectively eliminated untransfected SKOV3 cells within 2 weeks, ensuring selection of stably transfected clones. G418 (1 mg/mL) was applied for the selection of the stably transfected cells, a process that took two weeks.

The primer sequences for CNKSR1 are shown in Supporting Table 1.

The shRNA primers for CNKSR1 are shown in Supporting Table 2.

### 2.3. Patient Tissue Samples

The patients included were actively recruited for this study. The first review of the patients’ hospital records was conducted on January 1, 2024. From January 5, 2024, to December 25, 2024, 26 pairs of paraffin‐integrated samples of OC tissue and the contralateral normal ovarian tissue were collected from Jiaxing MCH. None of the selected patients had undergone any radiotherapy, chemotherapy, or any specialized therapy prior to surgery. The classification of tumor differentiation was tested according to the International Federation of Gynaecology and Obstetrics (FIGO) staging method classification standards. Every patient was staged in line with the 9^th^ edition of the Union International Cancer Control (UICC) Tumor‐Node‐Metastasis (TNM) SYSTEM OF staging.

Following Helsinki’s Pronouncement, the researcher gathered informed consent from all patients whose tissues were used in this study. Jiaxing MCH’s Ethics Committee of Jiaxing approved the study (Acceptance Number: KY‐2023‐230).

### 2.4. Quantitative Reverse Transcription Polymerase Chain Reaction (qRT‐PCR)

TRIzol reagent (Invitrogen, Camarillo, CA, USA) was used to extract the total RNA, and the PrimeScript kit (TaKaRa, Dalian, China) was utilized to obtain the corresponding DNA (cDNA). SYBR Green‐based qRT‐PCR amplification of cDNA was conducted through a Bio‐Rad CFX 96 actual‐time PCR arrangement. The reaction outcomes were characterized as initial denaturation at 95°C (30 s), then 40 rotations at 95°C (5), 55°C (30), and 72°C (30 s). β‐actin was applied as an internal control for mRNA quantification, and the 2^−ΔΔCt^ formula was used to evaluate CNKSR1’s relative expression.

The primers for *CNKSR1* and β‐actin are shown in Supporting Table 3.

### 2.5. Immunohistochemistry (IHC)

The paraffin‐implanted, formalin‐anchored ovarian tissue and dewaxing and rehydration were performed on the contralateral normal OC tissue segments. Activity involving endogenous peroxidase was inhibited by incubating the segments with 3% H_2_O_2_ (for 30 min). For antigen retrieval, citrate buffer (pH = 6) was applied to pretreated the section which was accomplished using pressure cooker‐mediated heating for 30 min, then incubated overnight (at 4°C) using rabbit polyclonal anti‐CNKSR1 antibody (1:100 dilution, Thermo Fisher Scientific, Waltham, MA, USA). Subsequently, following the washing process, the sections were incubated with a secondary antibody (1:4000 dilution, Abcam, Cambridge, UK) over 1 h and then stained with 3,3′‐diaminobenzidine tetrahydrochloride. Hematoxylin was employed to counterstain the sections, while a microscope was applied for observation. The results were recorded and analyzed for antigen expression using ImageJ. IHC staining was performed on all 26 pairs of samples, and representative images from six randomly selected pairs are shown in Figure 1(d). RS scoring: intensity (0–3) × positive cell percentage (0–4), total score (0–12) for quantifying IHC results.

FIGURE 1Differential expression of *CNKSR1* in ovarian cancer (OC) tissues. (a) The difference in CNKSR1 mRNA levels in OC cancer tissues and contralateral normal ovarian tissues was detected by RT‐PCR. (b, c) The difference in *CNKSR1* protein expression between ovarian cancer tissue and contralateral normal ovarian tissue was analyzed by Western blot. “T” stands for OC tissues; “N” stands for normal tissues. (d) The variance in *CNKSR1* expression among ovarian cancer tissues and contralateral normal ovarian tissues was detected by immunohistochemistry using an anti‐*CNKSR1* antibody (× 200). (e) Comparison of the *CNKSR1* protein levels in two groups, ^∗∗∗^ *p* < 0.001.

**Figure:**
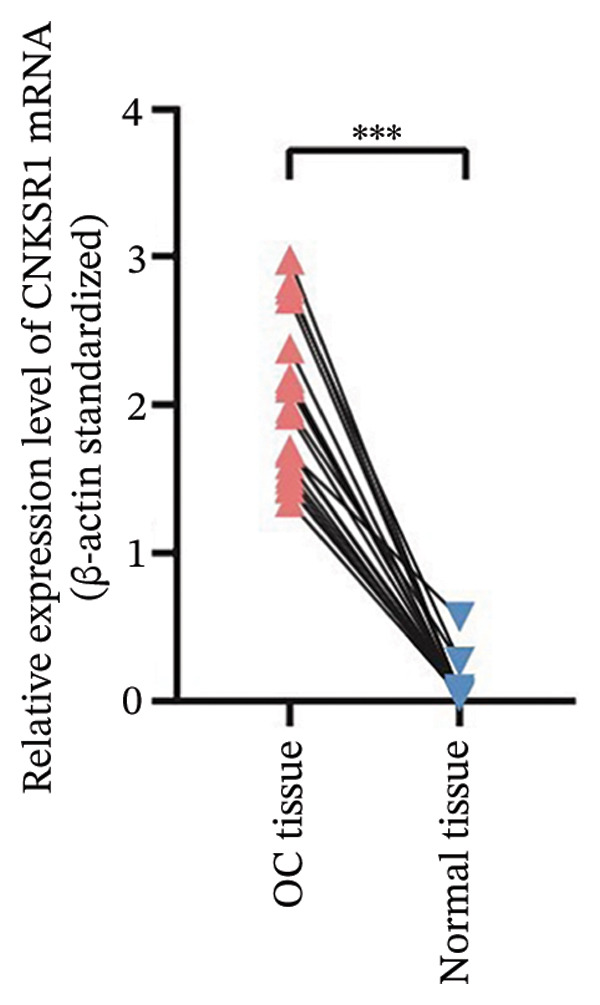
(a)

**Figure:**
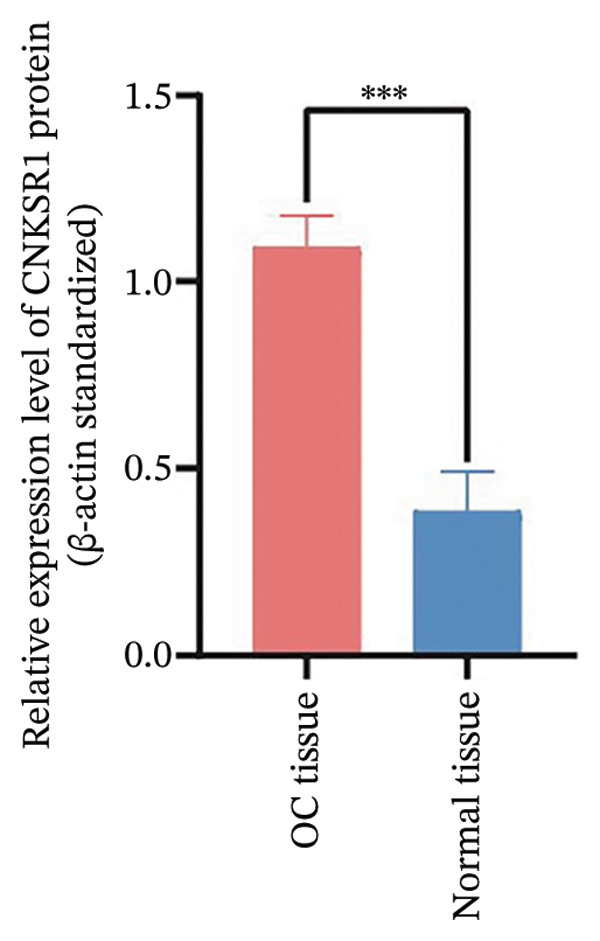
(b)

**Figure:**
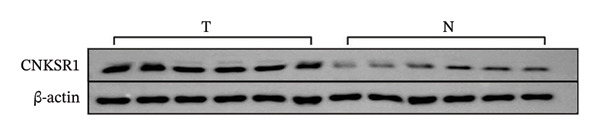
(c)

**Figure:**
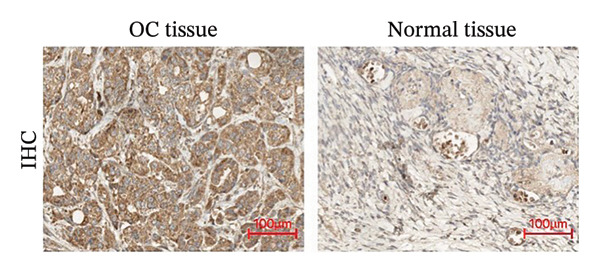
(d)

**Figure:**
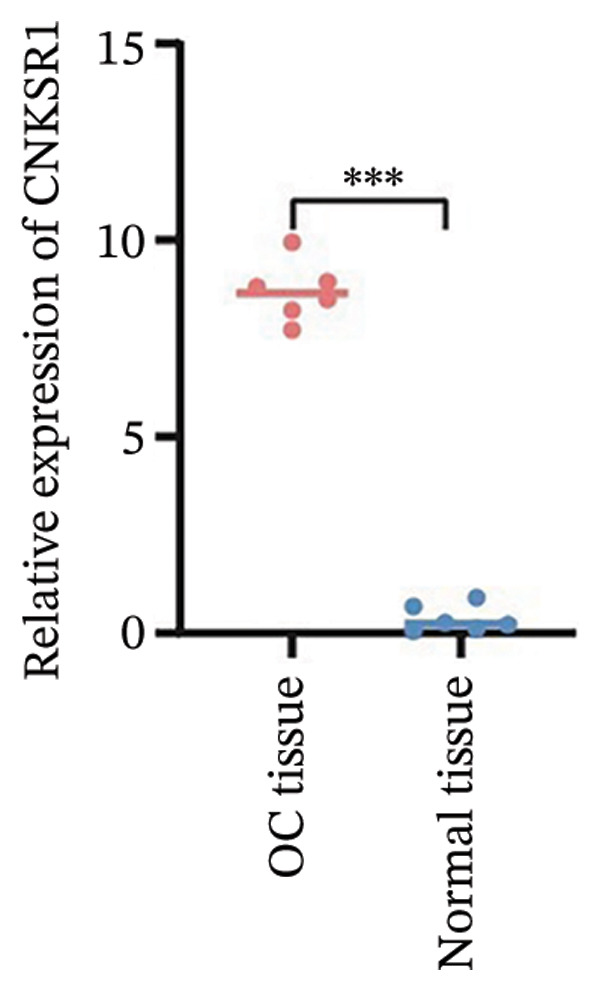
(e)

### 2.6. Western Blot

Utilizing RIPA lysis buffer, all proteins were obtained from OC cells, and the BCA assay was used to measure the quantity of protein. 8% SDS‐PAGE gels for ZEB1 and E‐cadherin and 12% gels for β‐actin were used to load 40 μg of protein and moved to membranes of polyvinylidene difluoride (PVDF). Following blockage of nonspecific binding with 2% bovine serum albumin, an overnight incubation of the membranes at 4°C was conducted with the main antibodies outlined as E‐cadherin (1:1000, rabbit anti‐E‐cadherin, anti‐CNKSR1 (1:1000), Abcam, Cambridge, UK), β‐catenin (1:2000, rabbit anti‐N‐cadherin, Abcam, Cambridge, UK), N‐cadherin (1:2000, rabbit anti‐Vimentin, Abcam, Cambridge, UK), Vimentin (1:2000, rabbit anti‐Vimentin, Abcam, Cambridge, UK), Snail (1:1000, rabbit anti‐zeb1, Abcam, Cambridge, UK), ZEB1 (1:1000, rabbit anti‐zeb1, Abcam, Cambridge, UK), and β‐actin (1:4000, mouse anti‐β‐actin, TransGene, Beijing, China). Following the washing with Tris‐buffered saline with Tween 20, incubation was done on the membranes using a secondary antibody (1:4000, Abcam, Cambridge, UK) at room temperature (1 h). Moreover, the enhanced chemiluminescence (ECL) kit helped in the visualization. Protein expression levels were quantified using ImageJ software and normalized to β‐actin.

### 2.7. CCK‐8 Assay

The CCK‐8 assay involved the collection of transfected SKOV3 cells, which were subsequently prepared as a cell suspension at a volume of 2 × 10^4^ cells/mL. The suspension of the cell (100 μL per well) was introduced into 96‐well plates. At 0, 24, 48 h, 10 μL of CCK‐8 reagent (Beyotime Biotechnology, Shanghai, China) was integrated into each well (6 replicates per group). The absorbance at 450 nm was calculated following a 2 h incubation at 37°C to evaluate cell proliferation.

### 2.8. EdU Assay

Following the instructions of the assay kit’s EdU cell propagation, Thermo Fisher Scientific (Shanghai, China), cells were seeded in a 24‐well plate and allowed to adhere for 24 h. Following was the addition of 300 μL of culture medium containing 1 × EdU into all wells, and 2 h of incubation of the cells was conducted. The medium was disposed, and a 50 min washing of cells in PBS was carried out twice. This was followed by the addition of 150 μL of 4% paraformaldehyde into the wells to fix the cells and incubate the cells at room temperature in 30 min. With the fixative removed, 150 μL of glycine (2 mg/mL) was placed into each well, and after that, the wells were incubated over a shaker during 5 min in order to neutralize the excess amount of paraformaldehyde. The solution of glycine was removed and the wells were washed with 0.5M PBS 5 min later, 300 μL of 0.5% Triton X‐100 permeabilization solution was added in each well and left at room temperature for 10 min. After the surface was washed with PBS (5 min) at the end of the permeabilization solution using PBS, the 100 μL of prepared staining reaction solution was placed in each of the wells and it covered the cells. The plate was left to incubate in the dark and at room temperature in 30 min. The staining reaction solution was disposed, and 2 or 3 times of the cells were washed with 0.5% permeabilization solution of Triton X‐100. The permeabilization solution and twice washing in 5 mL of PBS followed, 3000L of 1x Hoechst staining solution was added to each well, and the samples were incubated in the dark at room temperature (20–30 min). The staining solution was finally disposed of and the cells were washed twice using 300 μL of PBS. Images were then captured immediately using a fluorescence microscope (DMi8, Leica, Germany) at 400x magnification.

### 2.9. Wound Healing Assay

Cells that were transfected were plated in 6‐well plates at a density of 1 × 10^6^ cells per well and allowed to incubate overnight. A scratch was created in the cell monolayer using a sterile 200 μL pipette tip, followed by two washes with PBS. Subsequently, cells were cultured for a duration of 24 h in a serum‐free medium. Under microscopic observation, the wound closure area was examined, and images were taken at 50x magnification at both 0 and 24 h to assess the distance of migration. Wound width was measured at three random positions per well using ImageJ, and wound closure rate was calculated as (initial width–24 h width)/initial width × 100%.

### 2.10. Transwell Migration and Invasion Assays

#### 2.10.1. Migration Assay

SKOV3 cells were harvested and gathered as a suspension of serum‐free cells at a concentration of 1 × 10^6^ cells/mL. The cell suspension (100 μL) was inserted into the upper chamber of a Transwell insert. This lower chamber had 500 μL of 20% serum medium. The 48 h of incubation was followed by the fixation of cells using formaldehyde and staining with 0.1% crystal violet, washing cells (3 times) with PBS, and image acquisition.

#### 2.10.2. Invasion Assay

Treated SKOV3 cells were taken and made as a serum‐free cell suspension with a concentration of 1 × 10^6^ cells/mL. One hundred and 50 μL of a cell suspension was put in the top chamber of a Transwell insert which was covered with 1:8 Matrigel, M&G Biotechnology (Xiamen, China), to mimic the extracellular matrix density in vivo, as validated in preliminary experiments. A lower chamber held 500 μL of medium which had 20% serum. Following 48 h of incubation, cells were fixed using formaldehyde, stained using 0.1% crystal violet, washed thrice with PBS, and imaged.

### 2.11. Transcriptomic Sequencing and Bioinformatics Analysis

Overall RNA was obtained from the cancerous and contralateral normal ovarian tissues by an RNA extraction cassette (Promag, Shanghai, China). The extracted RNA samples were sent to Genewiz (Suzhou, China) for transcriptomic sequencing. Total RNA integrity was verified using an Agilent 2100 Bioanalyzer, with RIN ≥ 7.0 required for sequencing. Sequencing platform is Illumina NovaSeq 6000 and sequencing depth is 150 bp paired‐end reads, ∼6 Gb per sample. The differential gene analysis on the transcriptomic data were performed applying the R package “limma” to determine differentially expressed genes (DEGs). Two machine learning algorithms, LASSO and random forests (RFs), were applied to filter the DEGs. LASSO regression was performed with λ selected via 10‐fold cross‐validation, RFs were run with 1000 trees and ntree = 1000. Enrichment analysis and transcription factor predictions for the *CNKSR1* gene were performed through the STRING database (https://cn.string-db.org/). DAVID was employed for Gene Ontology (GO) and Kyoto Encyclopedia of Genes and Genomes (KEGG) analysis of the genes (*p* < 0.05, Count ≥ 2). Additionally, two online databases, ChEA3 (https://maayanlab.cloud/chea3/) and hTFtarget (https://bioinfo.life.hust.edu.cn/hTFtarget#!/) were employed to anticipate transcription factors targeting the *CNKSR1* gene.

### 2.12. Statistical Analysis

Differential expression analysis of DEGs was performed using R software (v4.4.1) with the ‘limma’ package; statistical analysis of cell experiments and clinical samples was conducted using GraphPad Prism 8. Typically, disseminated continuous data were presented as the mean ± standard deviation (x ± s). Paired sample *t*‐tests were employed for relating the two groups, while one‐way analysis of variance (ANOVA) was adopted for comparing various groups. For non‐normally distributed or inhomogeneous variance data, the median and interquartile ranges were reported. Comparisons between two groups were conducted using the Wilcoxon rank‐sum test (Mann–Whitney *U* test), and multiple group comparisons (between overexpression, knockdown, and control groups) were performed through the Kruskal–Wallis rank‐sum test (KW test). One‐way ANOVA followed by Bonferroni’s post hoc test. Non‐normally distributed data are presented as medians (interquartile range, IQR). All tests were two‐tailed, and *p* < 0.05 was considered statistically significant.

## 3. Results

### 3.1. Baseline Patient Information

As Table 1 illustrates, the average age of OC patients was 52 years (ranging between 34 and 65 years). Based on the results of histological assessment, 57.7% of patients were diagnosed with serous OC, 23.1% with mucinous OC, and 19.2% had other types of OC, such as high‐grade slurries, severe endometrioid lesions, and all undifferentiated and malignant mixed mesodermal tumors. Most patients (84.6%) were diagnosed as the late stage (FIGO III + IV).

**TABLE 1:** Baseline information.

Characteristics	No. patients (%)
Age at surgery (years)	
≤ 47.6	11 (42.3)
> 47.6	15 (57.7)
Histological	
Serous	15 (57.7)
Mucinous	6 (23.1)
Others	5 (19.2)
Histological grade	
G1	3 (11.5)
G2	14 (53.8)
G3	9 (34.6)
FIGO stage	
I	4 (15.4)
II	3 (11.5)
III + IV	19 (73.1)
TNM stage	
T1‐T2	8
T3‐T4	18
N0	10
N1	16
M0	22
M1	4

### 3.2. Identification of Key Differential Gene *CNKSR1*

The differential analysis of the transcriptomic data identified 78 genes that were downregulated and 101 genes that were upregulated in OC compared to the normal tissues. The top 10 DEGs included *CNKSR1, CT45A5, ALDH1A3, COL1A1, TIMP3, KCNS3, IGHV3-72, GPC4, IGLV3-27,* and *PAICS* (Figure 2(a)). Subsequently, two machine learning algorithms, LASSO and RFs, were applied to screen the 179 DEGs. The LASSO algorithm identified four key genes (Figure 2(b)), and the RF algorithm identified the top 40 most important genes (mean decrease accuracy > 1) (Figure 2(c)). By intersecting the 179 significant DEGs with the four key genes identified by the two algorithms, *CNKSR1* was ultimately identified as a key DEG (Figure 2(d)).

FIGURE 2Differential analysis. (a) Expression heatmap of the top 10 most significantly differentially expressed genes; (b) LASSO regression model identified key differential genes in ovarian cancer (OC); (c) random forest (RF) screening model identified key differential genes in OC; and (d) venn diagram of intersection genes between the LASSO regression and RF models and differentially expressed genes (DEGs).

**Figure:**
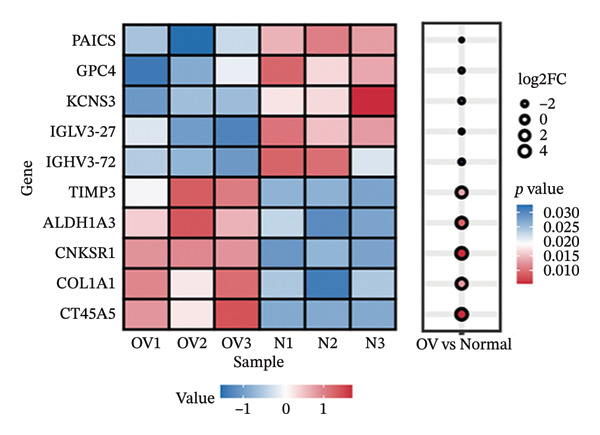
(a)

**Figure:**
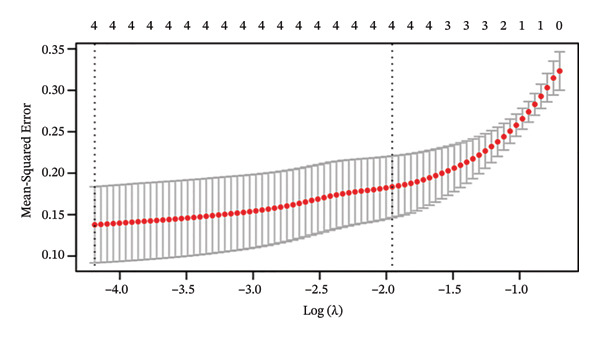
(b)

**Figure:**
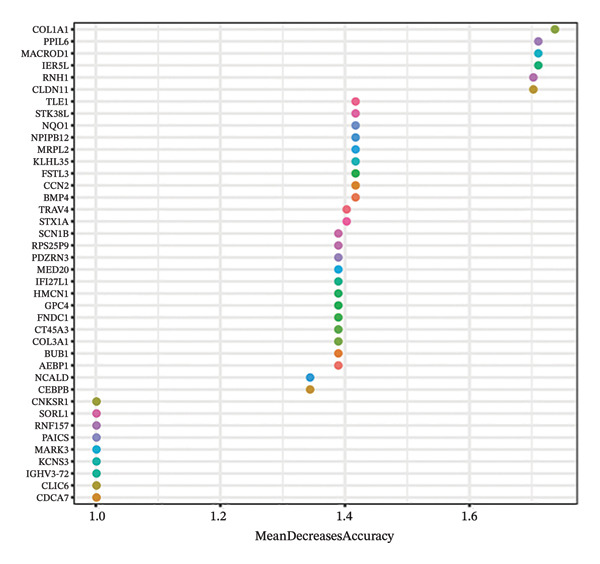
(c)

**Figure:**
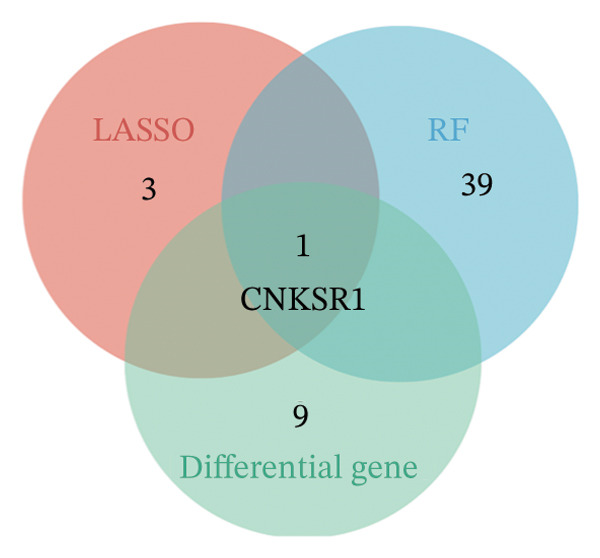
(d)

### 3.3. *CNKSR1* in OC Tissues

To examine the differential manifestation of *CNKSR1* in OC tissues, qRT‐PCR was conducted on 26 pairs of OC and contralateral normal ovarian tissues. The outcomes indicated that expression of *CNKSR1* mRNA in OC tissues was considerably greater than that in contralateral normal ovarian tissues (*p* < 0.001) (Figure 1(a)). Additionally, Western blot and IHC were conducted to detect the protein expression of *CNKSR1* in 6 pairs of OC and contralateral normal ovarian tissues. The outcomes of the Western blot analysis (Figures 1(b) and 1(c)) showed that the protein expression of *CNKSR1* in OC tissues was significantly higher than that in contralateral normal ovarian tissues (1.12 ± 0.15:1,*p* < 0.001). These results were further confirmed by IHC with an anti‐*CNKSR1* antibody (Figures 1(d) and 1(e)). However, the expression of *CNKSR1* was not found to be related to patient age.

### 3.4. Expression of *CNKSR1* in OC Cell Lines

*CNKSR1* mRNA and protein expression levels were assessed in OC cell lines (A2780, OVCAR3, SKOV3, and Hey) and human normal ovarian epithelial cells (ISOE80) by qRT‐PCR and Western blot, respectively. The outcomes exhibited that *CNKSR1* mRNA was significantly upregulated in OC cell lines compared with normal ovarian epithelial cells (ISOE80). Specifically, in SKOV3, A2780, and Hey cells, *CNKSR1* mRNA expression levels were elevated, with SKOV3 showing the highest increase. In contrast, OVCAR3 cells exhibited a lower *CNKSR1* expression level (Figures 3(a), 3(b), 3(c)). This discrepancy may reflect subtype‐specific expression of *CNKSR1*, which indicates that the therapeutic effect of *CNKSR1*‐targeted therapy may vary based on different subtypes of OC. Subsequently, the SKOV3 cell line was selected for cellular experiments.

FIGURE 3The expression of *CNKSR1* in OC cell lines. (a) Analysis of *CNKSR1* mRNA expression by RT⁃PCR in OC and normal ovarian tissue cell lines. (b, c) Differential protein expression of *CNKSR1* in ovarian cancer tissues and adjacent tissues was detected by Western blot; ns: *p* > 0.05, ^∗∗^ *p* < 0.01, ^∗∗∗^ *p* < 0.001.

**Figure:**
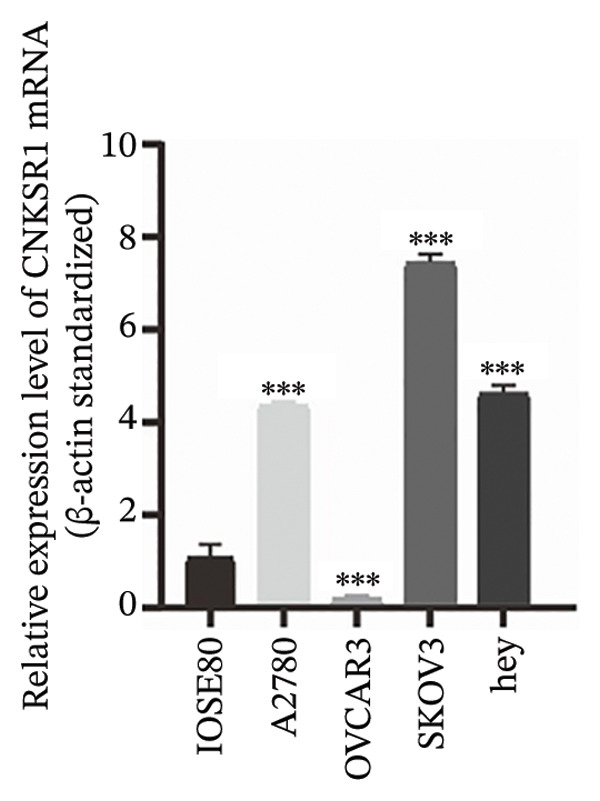
(a)

**Figure:**
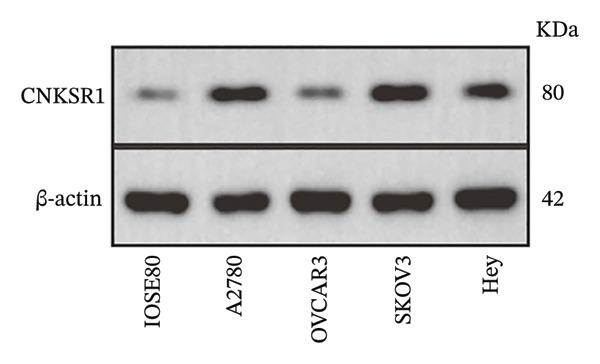
(b)

**Figure:**
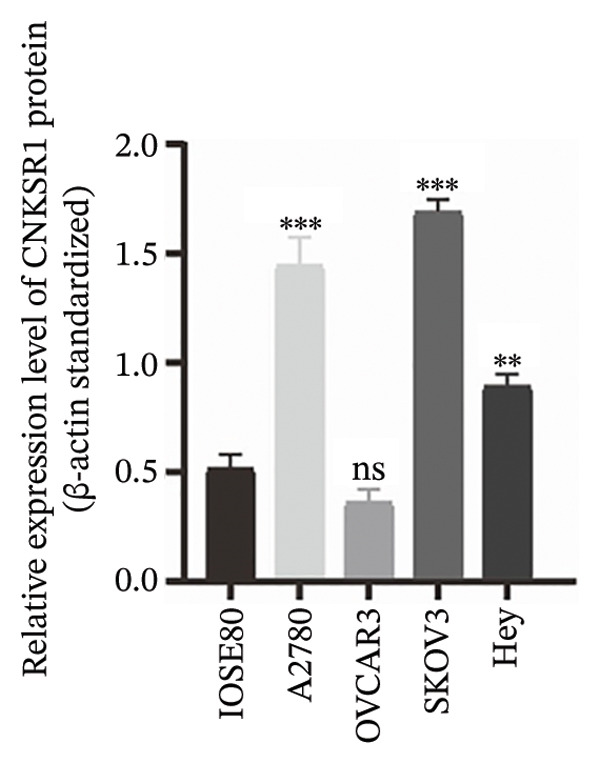
(c)

### 3.5. Correlation of *CNKSR1* With Proliferation, Migration, and Distant Metastasis of OC

To investigate the biological significances and signaling trajectories potentially associated with the *CNKSR1* gene, the STRING database was used to identify 50 proteins co‐expressed with *CNKSR1*, and a PIP network was constructed (Figure 4(a)). The DAVID was adopted to conduct enrichment examination on these 50 genes. GO analysis indicated that *CNKSR1* expression might be associated with cell migration‐related functions (Figure 4(b)). Subsequently, the functional state of *CNKSR1* in OC was explored using the CancerSEA database. The results demonstrated that *CNKSR1* mRNA expression was positively interconnected with OC differentiation, angiogenesis, and metastasis and negatively connected with DNA repair, cell cycle, and DNA damage (Figures 4(c) and 4(d)).

FIGURE 4Correlation of *CNKSR1* levels with proliferation, migration, and distant metastasis of OC. (a) Diagram of the protein interaction network of the *CNKSR1* protein. (b) GO enrichment analysis of the *CNKSR1* gene. (c, d) Functional analysis of the *CNKSR1* protein, ^∗^ *p* < 0.05, ^∗∗^ *p* < 0.01, ^∗∗∗^ *p* < 0.001.

**Figure:**
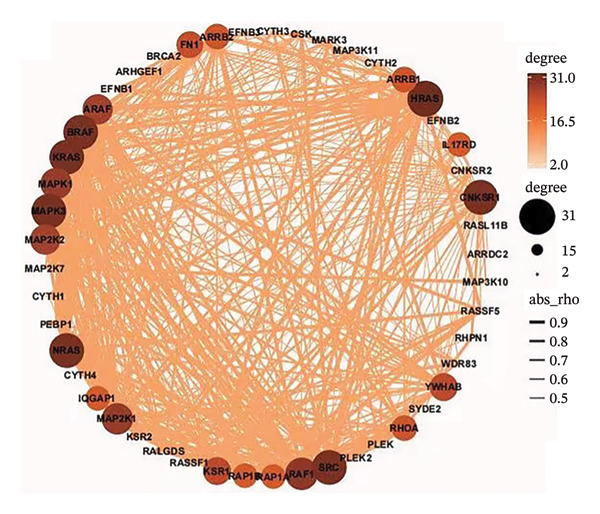
(a)

**Figure:**
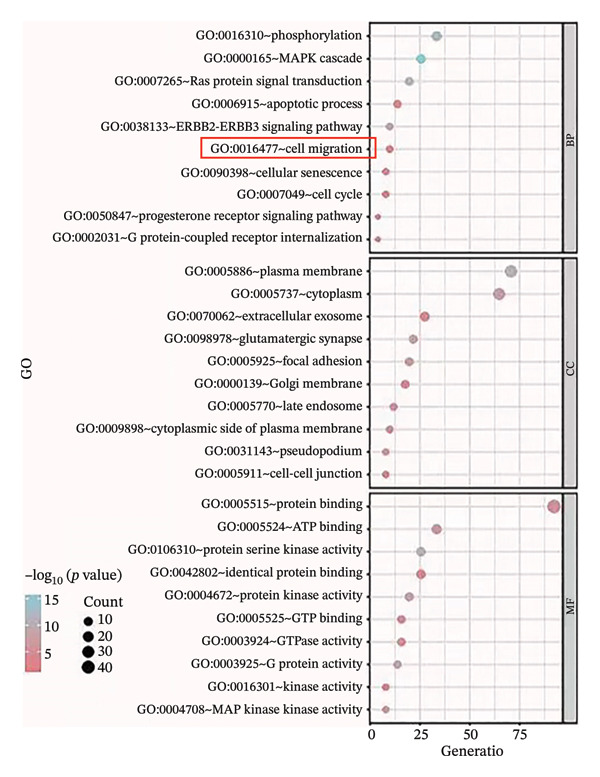
(b)

**Figure:**
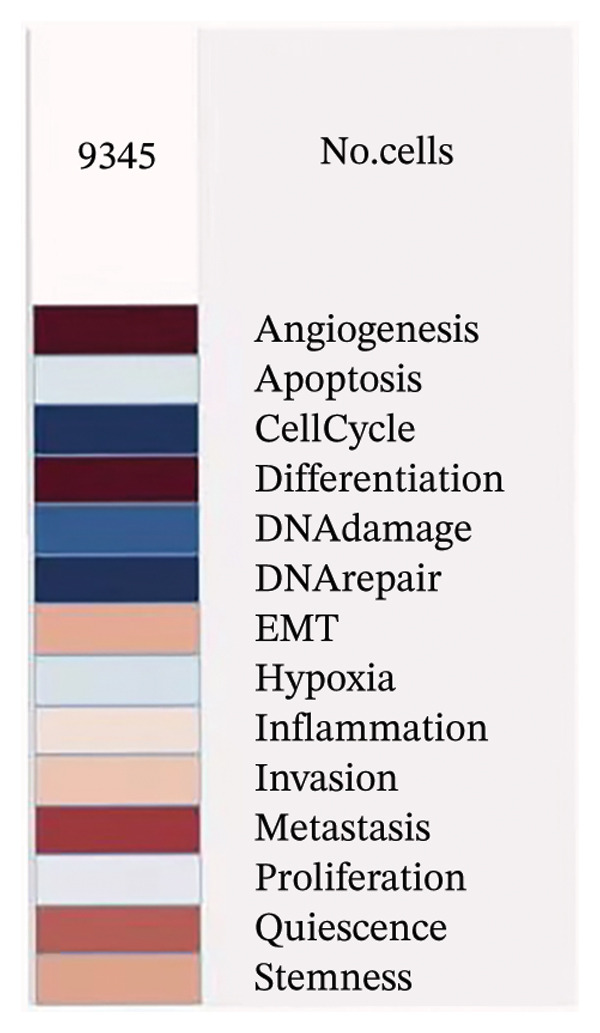
(c)

**Figure:**
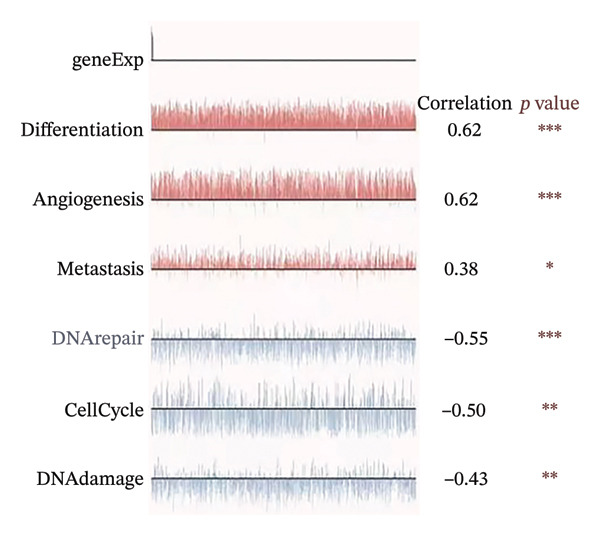
(d)

### 3.6. *CNKSR1* Accelerates OC Cell Migration and Invasion

The expression of *CNKSR1* in SKOV3 cells was reduced by transfecting the cells with shRNA. The highest knockdown efficiency was observed in cells transfected with sh‐*CNKSR1*‐1 (*p* < 0.001) (Figure 5(a)). Significant *CNKSR1* overexpression in SKOV3 cells was achieved by transfecting the cells with the plenti‐*CNKSR1* and OV‐*CNKSR1*‐1 (*p* < 0.001) (Figure 5(b)). Functional assays were then conducted. As indicated by the CCK8 assay, *CNKSR1* knockdown significantly inhibited the proliferation of OC cells, whereas overexpression of *CNKSR1* was associated with markedly increased proliferation (*p* < 0.001) (Figure 5(c)). Similarly, compared with the control, the percentage of EdU‐positive proliferating cells in the *CNKSR1* knockdown group was significantly lowered. At the same time, the overexpression of *CNKSR1* led to a significantly higher percentage of EdU‐positive cells (Figures 5(d), 5(e), 5(f)).

FIGURE 5
*CNKSR1* promotes the proliferation and migration abilities of ovarian cancer cells. (a) Knockdown efficiency of *CNKSR1* in the SKOV3 cell line measured by RT‐PCR. (b) The overexpression efficiency of *CNKSR1* in the SKOV3 cell line was measured by RT‐PCR. (c) CCK⁃8 assay was performed to detect the effect of *CNKSR1* on the proliferation ability of SKOV3. (d–f) EdU (5‐ethynyl‐2′‐deoxyuridine) assay was performed to detect the effect of *CNKSR1* on the proliferation of SKOV3. (g) A migration assay was performed to analyze the effect of *CNKSR*1 on the migration ability of SKOV3 cells, with ns indicating *p* > 0.05 and ^∗∗∗^ *p* < 0.001.

**Figure:**
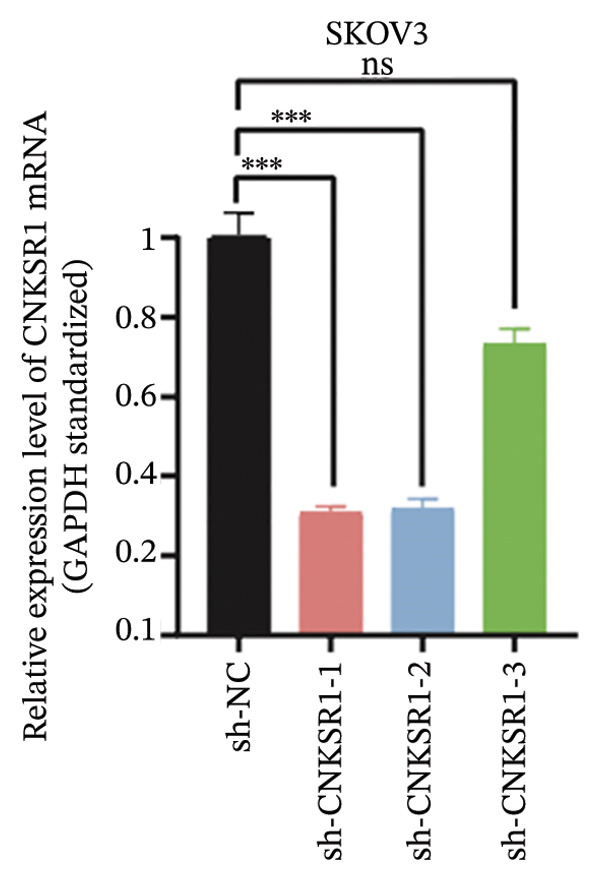
(a)

**Figure:**
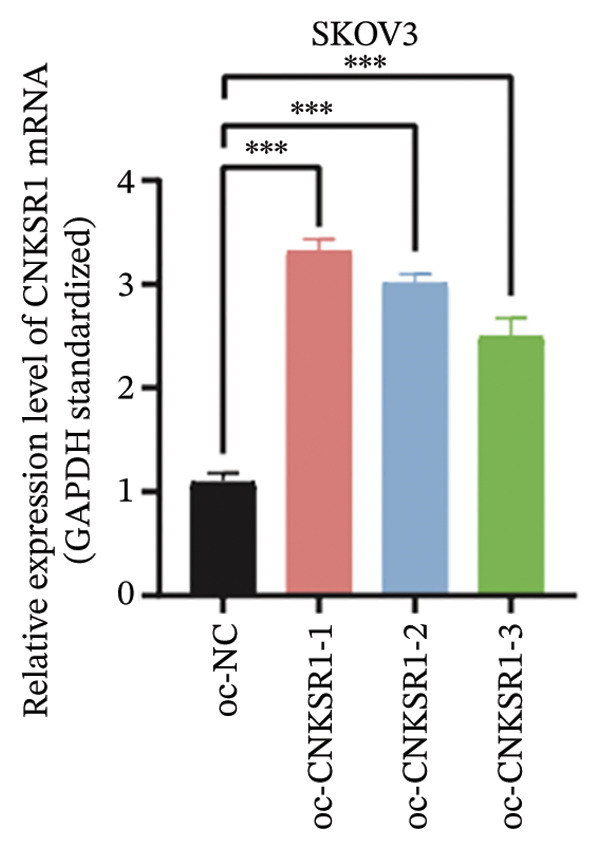
(b)

**Figure:**
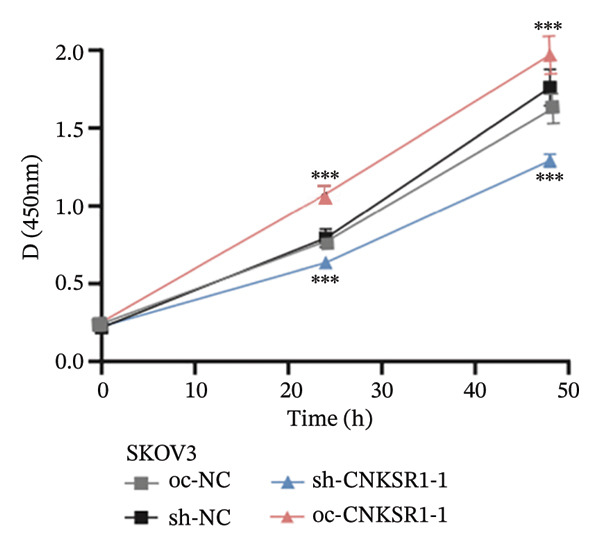
(c)

**Figure:**
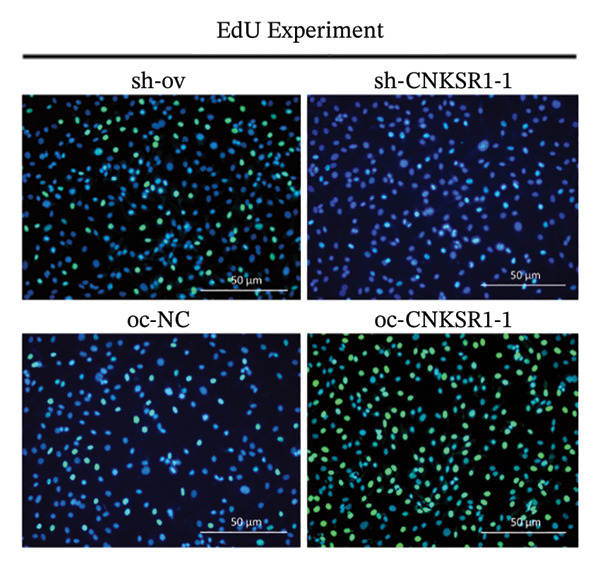
(d)

**Figure:**
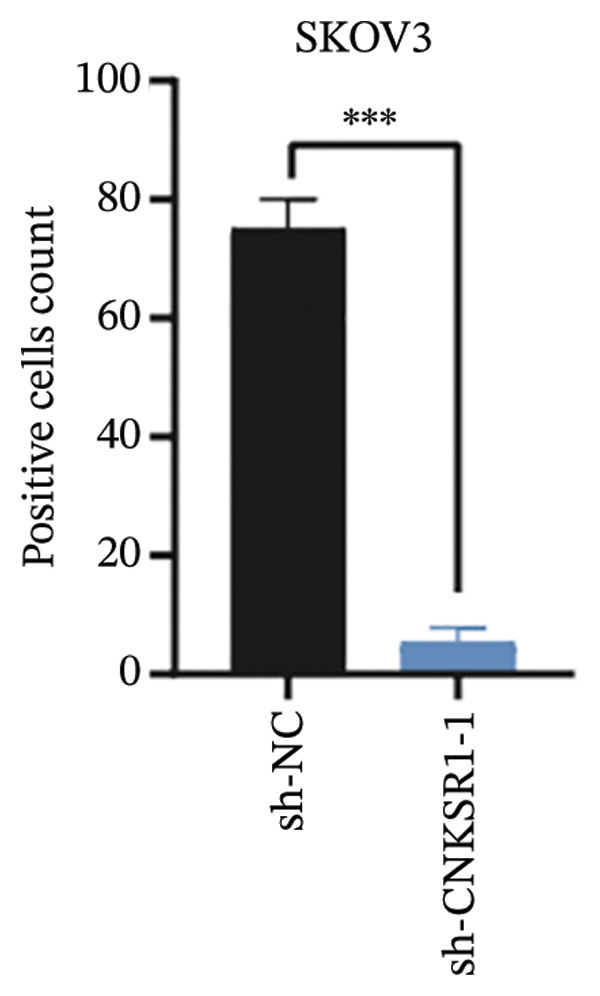
(e)

**Figure:**
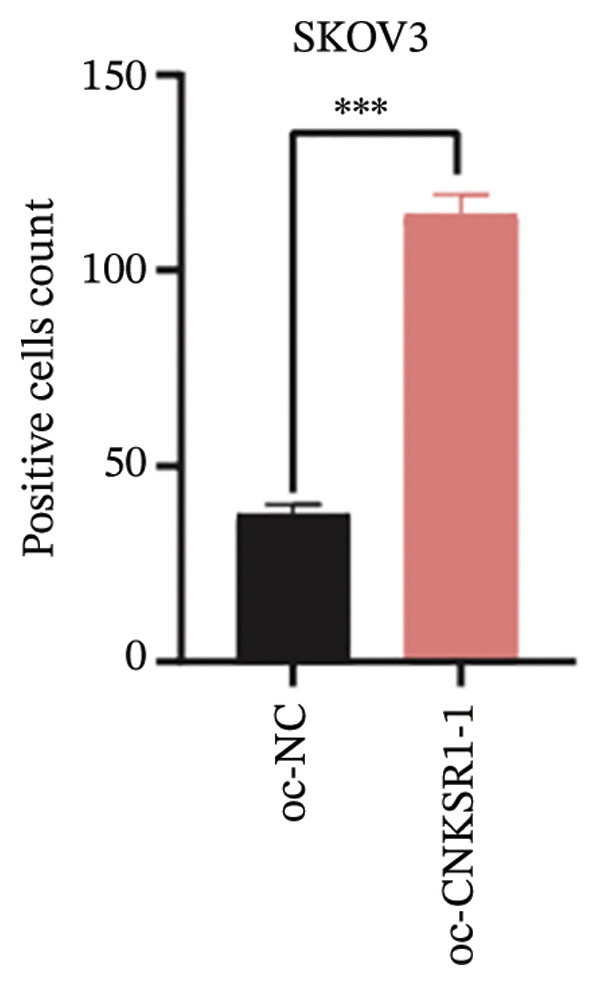
(f)

**Figure:**
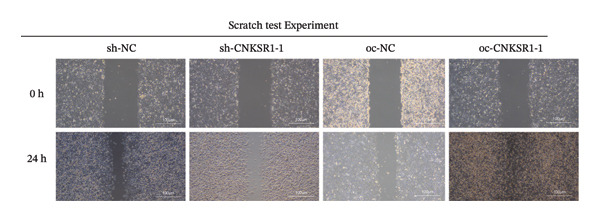
(g)

The wound healing assay further confirmed that *CNKSR1* knockdown substantially inhibited the migration of OC cells, while overexpression of *CNKSR1* markedly enhanced the migration (Figure 5(g)). The Transwell assays confirmed that *CNKSR1* knockdown suppressed OC cell migration and invasion, whereas the overexpression of *CNKSR1* significantly promoted these abilities. Collectively, these findings suggest that the overexpression of *CNKSR1* enhances the proliferation, migration, and invasion of OC cells, while *CNKSR1* knockdown reverses these effects (Figures 6(a), 6(b), 6(c), 6(d), 6(e), 6(f)).

FIGURE 6Effect of *CNKSR1* levels on the migration and invasion of OC cells. (a–c) Migration assay to analyze the effect of *CNKSR1* knockdown on the migration ability of OC cells SKOV3. (d–f) Invasion assay to analyze the effect of *CNKSR1* knockdown on the invasion ability of OC cells SKOV3, ^∗∗^ *p* < 0.01, ^∗∗∗^ *p* < 0.001.

**Figure:**
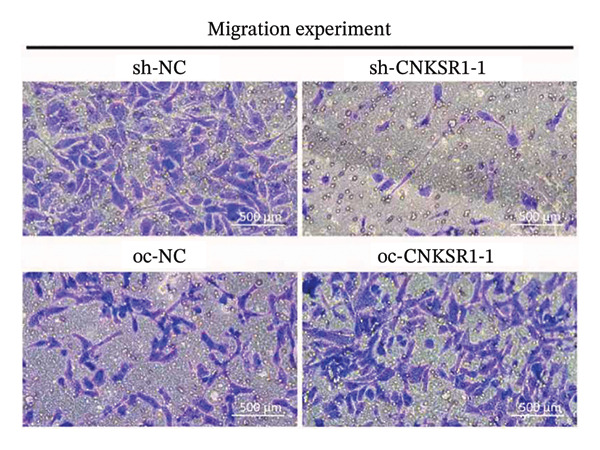
(a)

**Figure:**
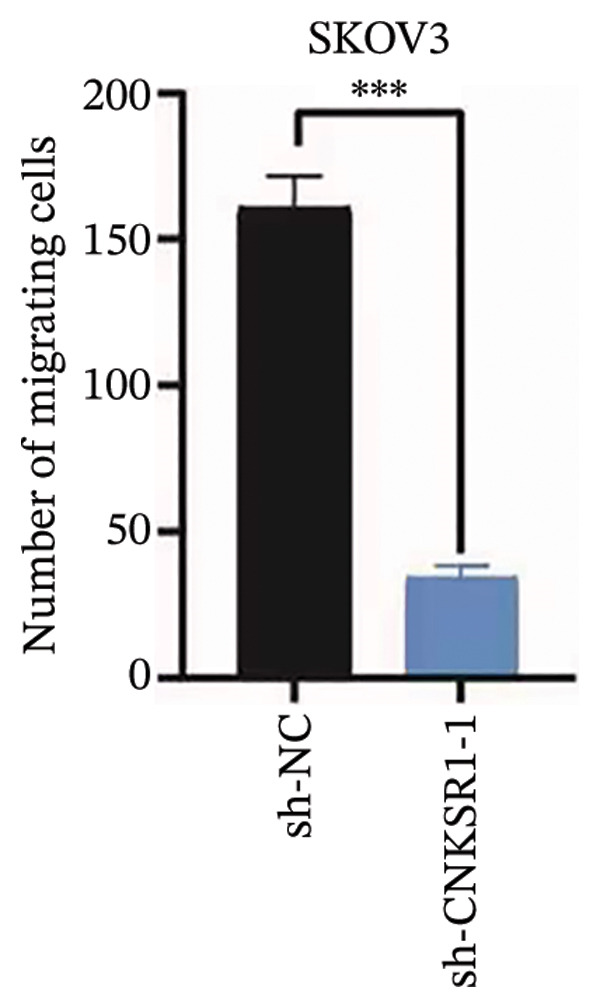
(b)

**Figure:**
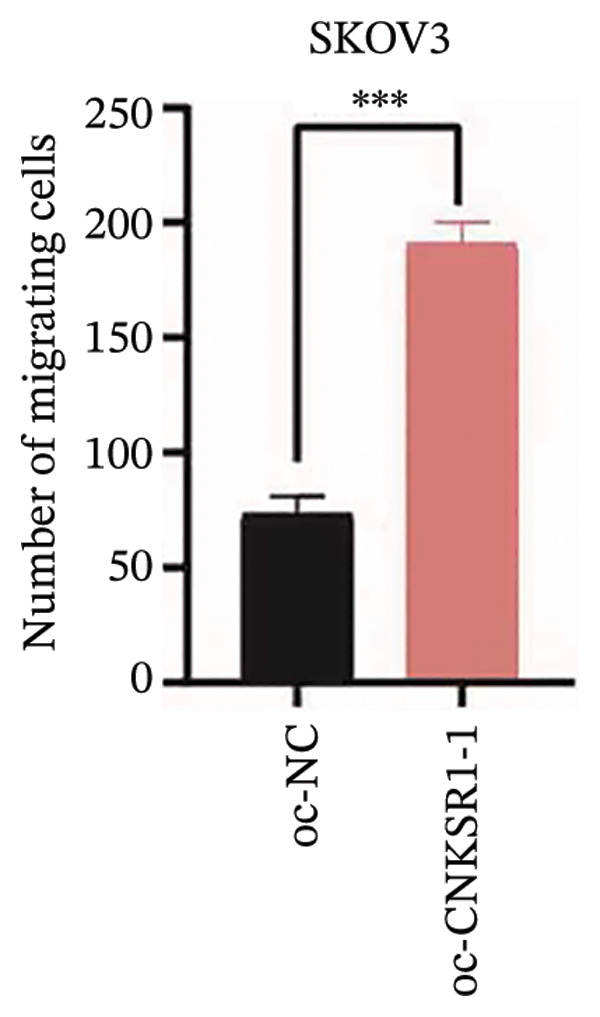
(c)

**Figure:**
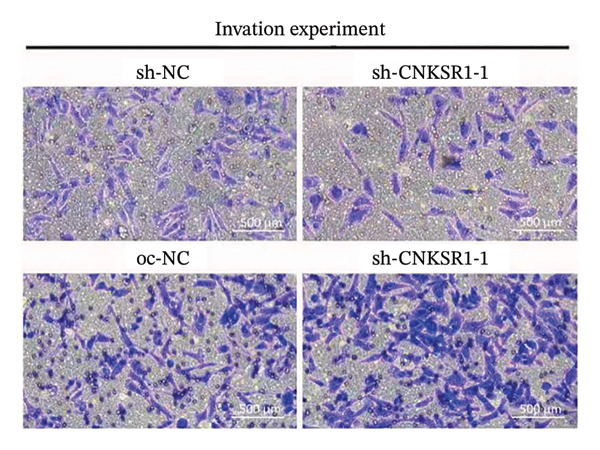
(d)

**Figure:**
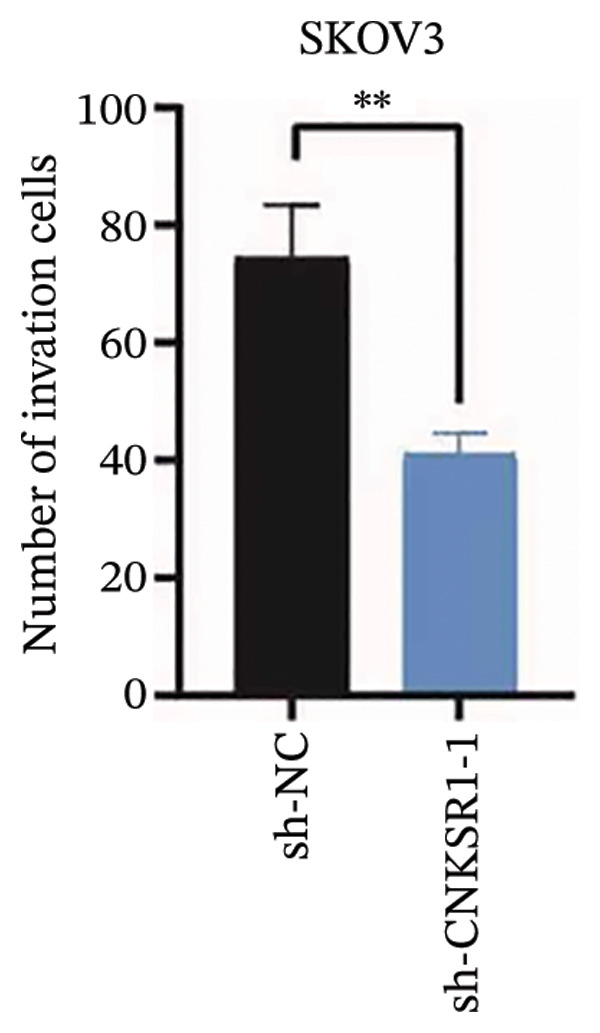
(e)

**Figure:**
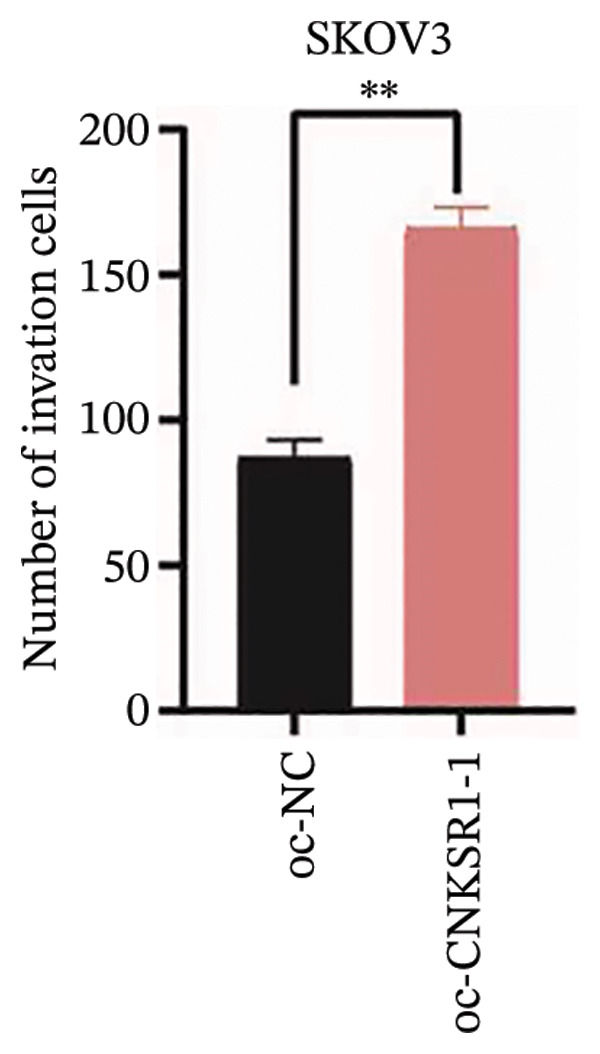
(f)

### 3.7. *CNKSR1* Induces EMT by Regulating Transcription Factors Snail/ZEB1

ChEA3 and hTFtarget databases were used to predict transcription factors associated with *CNKSR1* mRNA. The analysis identifies 14 transcription factors that may be potentially involved in *CNKSR1* regulation (Figure 7(b)), including *ZEB1, SPI1, NR2C2, TEAD4, TFAP2A, GATA3, YY1, CTCF, THAP1, GABPA, FOS, MAZ, ZBTB7A*, and *TCF7L2* (Figure 7(a)). Since EMT is a key mechanism in cell migration and invasion, the impact of *CNKSR1* on EMT progression was further explored.

FIGURE 7
*CNKSR1* alters the expression of epithelial‐mesenchymal transition (EMT)‐related proteins. (a) Diagram of the network of transcription factors that regulate CNKSR1 protein. (b) Venn diagram of intersection of transcription factors regulating CNKSR1 protein. (c, d) *CNKSR1* altered the expression of EMT‐related proteins as determined by Western blot, ^∗^ *p* < 0.05, ^∗∗^ *p* < 0.01, ^∗∗∗^ *p* < 0.001.

**Figure:**
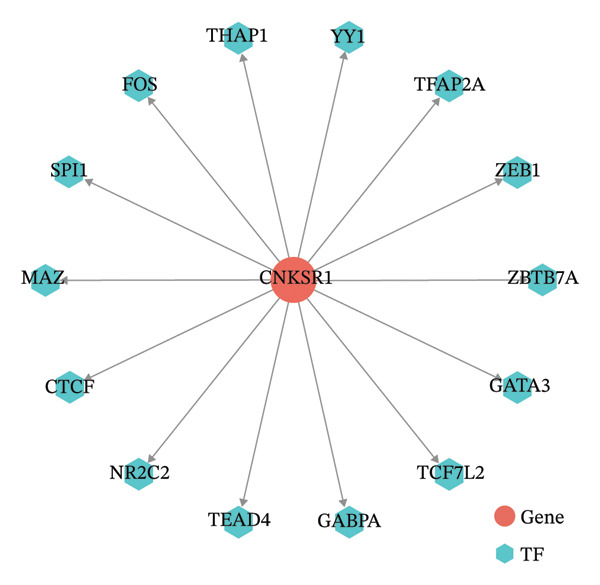
(a)

**Figure:**
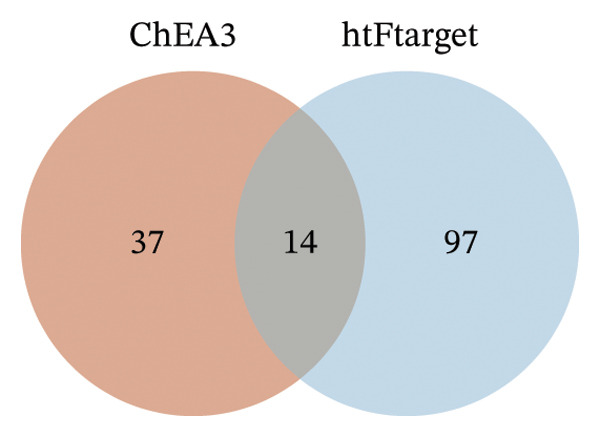
(b)

**Figure:**
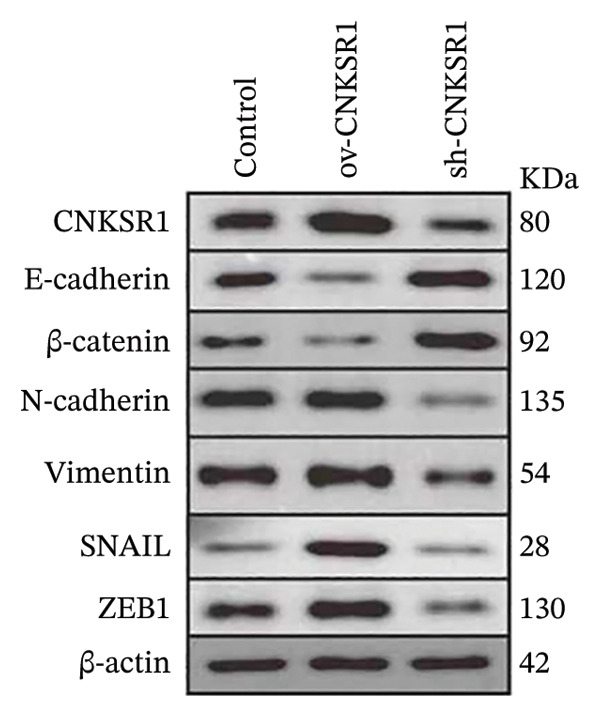
(c)

**Figure:**
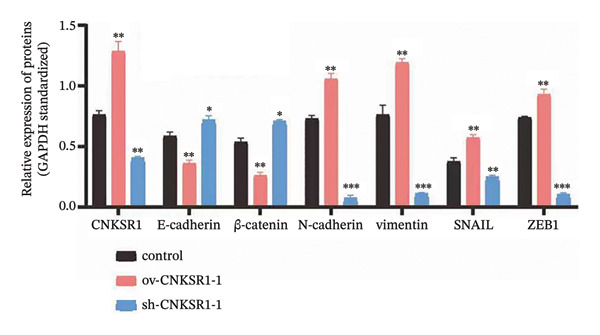
(d)

Western blot analysis was performed to detect the expression of epithelial markers (E‐cadherin and β‐catenin), mesenchymal markers (N‐cadherin and Vimentin), and EMT transcription factors (*Snail* and *ZEB1*) in SKOV3 cells. The results showed that the overexpression of *CNKSR1* in SKOV3 cells increased the expression of N‐cadherin, Vimentin, Snail, and ZEB1, while downregulating E‐cadherin and β‐catenin. In contrast, *CNKSR1* knockdown in SKOV3 cells upregulated the expression of E‐cadherin and β‐catenin, and downregulated the expression of N‐cadherin, Vimentin, Snail, and ZEB1 (Figures 7(c) and 7(d)).

## 4. Discussion

This study employed bioinformatics analysis, machine learning, and molecular biology methods to identify CNKSR1 as a protein extremely conveyed in OC tissues, playing a role in the proliferation, migration, and invasion of OC cells. The outcomes suggest that *CNKSR1* may induce the invasion and metastasis of OC cells by controlling the expression of EMT‐related proteins Snail and ZEB1. Consistent with Quadri et al. (2017), who reported *CNKSR1* as a poor prognostic marker in pancreatic cancer, our study demonstrates that *CNKSR1* is overexpressed in OC and promotes tumor progression. However, we are the first to identify that this pro‐tumor effect in OC is mediated by the *Snail/ZEB1*‐dependent EMT pathway, expanding our understanding of *CNKSR1’s* oncogenic mechanisms across different cancer types.

These results confirm previous studies that identified *CNKSR1* as a potential tumor biomarker, with elevated mRNA levels observed in pancreatic, lung, and breast cancers [1–4]. Furthermore, CNKSR1 was shown to be significantly upregulated in colorectal cancer cells. Its inhibition suppressed the growth of KRAS‐mutant colon cancer cells and disrupted the Raf/MEK/extracellular signal‐regulated kinase (ERK), Rho, and RalA/B signaling pathways [1, 3]. Quadri et al. [2] analyzed 120 pancreatic tissue samples and found that *CNKSR1* was highly portrayed in pancreatic cancer cells, and its high expression interrelated with poorer tumor differentiation. Similarly, Lee et al. [5] demonstrated that, compared with normal tissues and BRAFV600E‐negative thyroid cancers, Kinase Suppressor of RAS1 (KSR1) and CNKSR1 were significantly upregulated in thyroid cancer tissues, BRAFV600E‐positive thyroid cancers, and metastatic lymph node tissues, which was associated with enhanced Notch signaling. Together with the current study, these findings indicate that *CNKSR1* may serve a crucial role in the development and progression of tumors, promoting its capacity as a new biomarker for OC.

*CNKSR1* has been linked to cell proliferation, migration, invasion, and tumorigenesis (Fritz, Varga, and Radziwill, 2010; Cho et al., 2014; Fischer et al., 2017). In breast cancer cells, the overexpression of *CNKSR1* promotes cell proliferation. This process depends on the phosphatidylinositol 3‐kinase (PI3K) pathway, as the inhibition of PI3K by LY294002 abrogates *CNKSR1*‐induced proliferation [6]. Fischer et al. [7] also found that the overexpression of *CNKSR1* enhances cell proliferation and migration. *CNKSR1* links the activation of Ephrin B1 to the activation of C‐Jun N‐terminal kinase (JNK) via small GTPases, promoting cell migration. However, knocking down *CNKSR1* blocks EphrinB1‐mediated cell migration and JNK activation [1]. Fritz and Radziwill [8] reported that the knockdown of *CNKSR1* reduces the invasion of breast and cervical cancer cells, a process closely related to decreased expression of matrix metalloproteinase‐9 (MMP‐9) and membrane‐type 1 MMP (MT1‐MMP). They showed that overexpression of *CNKSR1* enhances MT1‐MMP promoter activity in an NF‐κB‐dependent manner, promoting invasion. *CNKSR1* was also shown to interfere with the processing of NF‐κB p100 to p52, affecting its proper nuclear localization and thereby inhibiting tumor cell invasion [9]. The expression of *CNKSR1* is related to the nuclear phosphorylation of ERK, suggesting that *CNKSR1* may affect the progression of pancreatic cancer by regulating related signal pathways (13). The study also found that the expression level of *CNKSR1* mRNA is negatively correlated with the expression of PD‐L1 mRNA and immune cell infiltration in lung adenocarcinoma (14). A recent study also shows that *CNKSR1* promotes the crosstalk of AKT signals through the scaffold function of phosphorylated AKT, thus playing a regulatory role in the adaptive resistance to MEK inhibition [10]. However, the biological role of *CNKSR1* in OC has not been reported. This study provides multiple lines of evidence confirming that *CNKSR1* is highly expressed in OC tissues and cells and overexpression of *CNKSR1* enhances proliferation, migration, and invasion of OC cells. At the same time, knockdown can reverse these effects, suggesting that *CNKSR1* acts as an oncogene in OC.

This study detected a variability in the *CNKSR1* expression in different OC cell lines. OC is a heterogeneous disease with several histologic subtypes, such as serous, endometrioid, mucinous, and clear cell carcinomas, with unique genetic and biological features and different clinical outcomes [11, 12]. In addition to various subtypes of OC, intratumoral heterogeneity, characterized by different cell populations within a lesion, has also been linked to the differences in the progression and ultimate lethality of OC [13]. In this study, SKOV3, which is a human ovarian adenocarcinoma cell line and a representative of the mesenchymal subtype, showed the highest expression of *CNKSR1*. In contrast, OVCAR3 cells, which represent an epithelial subtype, exhibited a lower expression level of *CNKSR1*. This subtype‐specific expression of *CNKSR1*, observed in the study, may have clinical significance, as it suggests that the effect of *CNKSR1*‐targeted therapy may vary across different subtypes of OC. The mesenchymal subtype of OC may be more sensitive to *CNKSR1*‐targeted treatment due to higher expression of the target molecule. In contrast, the sensitivity of the epithelial subtype of OC may be lower due to the lower expression of *CNKSR1*. Future studies of the heterogeneity between major histological types of OC may allow for a transition from a single therapeutic approach to a precise targeting of subtype‐specific characteristics.

The results of this study also showed that *CNKSR1* may induce OC invasion and metastasis by regulating transcription factors Snail and ZEB1 to mediate EMT. Invasion and metastasis are complex, multistep processes regulated by numerous genes, involving various behaviors of tumor cells, including adhesion, matrix degradation, migration, and angiogenesis, all of which are influenced by the regulation of multiple genes [14, 15]. Although many genes have been identified in tumorigenesis and metastasis, the precise molecular mechanisms remain unclear. EMT is a reversible developmental program exploited by cancer cells, allowing them to transition from an epithelial phenotype with apical‐basal polarity and cell–cell adhesion to a more invasive and motile mesenchymal state with a spindle‐like morphology [16, 17]. EMT not only enhances cellular motility and invasiveness but is also associated with tumor cells acquiring stem cell‐like characteristics, increased therapeutic resistance, and reduced immune response [16, 18, 19]. At the molecular level, EMT involves various changes, including decreased epithelial markers and increased mesenchymal markers. E‐cadherin, a key component of cell–cell adhesion, is a critical marker of EMT [20]. ZEB1, a key driver of EMT, also acts as a major suppressor of E‐cadherin. Snail, a member of the Snail family, inhibits the expression of E‐cadherin [21]. In xenograft models, the knockdown of E‐cadherin significantly suppresses the malignant phenotype and growth of mesenchymal OC cell lines [22, 23]. The overexpression of *CNKSR1* may upregulate Snail and ZEB1, thereby downregulating the expression of E‐cadherin and promoting EMT [24], which is further confirmed in this study. Our results showed that in SKOV3 cells, knockdown of *CNKSR1* significantly decreased the levels of N‐cadherin, Vimentin, Snail, and ZEB1 while increasing the expression of E‐cadherin and β‐catenin. Conversely, overexpression of *CNKSR1* significantly increased the levels of N‐cadherin, Vimentin, Snail, and ZEB1, while downregulating the expression of E‐cadherin and β‐catenin. These results suggest that *CNKSR1* may regulate the migration and invasion of OC cells through the EMT mechanism mediated by transcription factors Snail and ZEB1.

Also, various limitations are reflected in this study. Firstly, the limited sample size increases the risk of selection bias. More research with a greater sample size is required to justify the study’s findings, including various histological types. Secondly, this experiment lacks in vivo validation. It did not use a xenograft model to confirm the role of *CNKSR1* in metastasis. The expression of *CNKSR1* is not correlated with the prognosis of patients, such as overall survival rate and progression‐free survival rate. Limited cell line usages, only SKOV3, was used for functional assays—validate results in other OC cell lines to confirm generality. In addition, in vivo experiments have not yet evaluated the exact function and molecular mechanism of *CNKSR1* activity in OC, which could offer additional understanding into the role of *CNKSR1* in tumor growth, metastasis, and the host immune response. Future in vivo studies should focus on creating knockout animal models to examine the changes in the biological role of *CNKSR1* in tumor growth, metastasis, and angiogenesis, as well as host immune response. Future studies with follow‐up data of patients may validate the clinical significance of the results. No correlation between *CNKSR1* expression and patient prognosis (e.g., overall survival and progression‐free survival). Limited cell line usages, only SKOV3, was used for functional assays—validate results in other OC cell lines to confirm generality. Further research is needed to validate and expand our observations and confirm the value of *CNKSR1* as a potential therapeutic target for OC.

*CNKSR1* is an oncogene that is significantly overexpressed in OC tissues and cells. It promotes OC cell proliferation, migration, and invasion by regulating the Snail/ZEB1‐mediated EMT pathway. These findings suggest that *CNKSR1* may serve as a potential therapeutic target for OC, particularly in the mesenchymal subtype. However, further in vivo studies and large‐scale clinical validation are required to confirm its clinical utility. In addition, the design of a targeted therapy against *CNKSR1* might also be a challenge.

## Author Contributions

Weili Zhu, Yimin Huang, and Jianguo Wang: conceptualization, writing–original draft, and writing–review and editing. Jiayue Huang: writing–review and editing. Sijia Shen: conceptualization, methodology, formal analysis, writing–original draft, writing–review and editing.

## Funding

This work was supported by the Natural Science Foundation of Zhejiang Province (LQ21H160040) and Research on Technological Innovation of People’s Livelihood in Jiaxing (2022AD30086).

## Disclosure

The completed manuscript has been reviewed and authorized by all authors.

## Ethics Statement

The research was performed based on Helsinki’s Declaration and ratified by the IRB of the Ethics Committee of Jiaxing MCH (protocol code: KY‐2023‐230, approved on 29 December 2023).

## Consent

This was an observational study that analyzed de‐identified data; thus, patient consent was waived due to the retrospective nature of the study.

## Conflicts of Interest

The authors declare no conflicts of interest.

## Supporting Information

Supporting link: My Supporting table and the unaltered original Western Blot images are all available at the following link.

## Supporting information


**Supporting Information** Additional supporting information can be found online in the Supporting Information section.

## Data Availability

The employed and/or examined datasets in the current study can be requested at the corresponding authors on a reasonable basis. The sequencing results have been uploaded to the NBCI public database, with the identification number PRJNA1223134 (https://www.ncbi.nlm.nih.gov/search/all/?term=PRJNA1223134).
