# Supplementary material for: A Multiomics and Bioinformatics Analysis of the Role and Mechanism of CNKSR1 in Ovarian Cancer Progression
Source: Int J Genomics. 2026 Apr 11;2026:1546020. doi: 10.1155/ijog/1546020 (PMC13069470; doi:10.1155/ijog/1546020)
Supplement: Supplementary file 1 — Supporting Information Additional supporting information can be found online in the Supporting Information section. [file IJOG-2026-1546020-s001.zip › 细胞鉴定图.zip!/τ╗åΦâ₧Θë┤σ«Üσ¢╛/IOSE-80_Σ║║μ¡úσ╕╕σì╡σ╖óΣ╕èτÜ«τ╗åΦâ₧_TCH-C402_240103M201_A5.pdf]

成品检验报告书
Certificate of Analysis

产品名称 Product Name: **人正常卵巢上皮细胞（IOSE-80）**

产品货号 Cat. No. **TCH-C402**

产品批号 Lot. No. **240103M201**

检验依据 Standards: **现行版《中国药典》三部和企业内控标准**
The current version of the Chinese Pharmacopoeia & enterprise internal control standards.

检测项目<br>Content	检测方法<br>Method	检测标准<br>Specification	检测结果<br>Results
**生长特性**<br>**Growth properties**	常规培养<br>Conventional cultivation	贴壁细胞<br>Adherent	贴壁细胞<br>Adherent
**细胞活率**<br>**Post-freeze viability**	对复苏后的细胞采取台盼蓝染色计数法<br>Cell count using Trypan Blue stain method	≥80%	90.63%
**细胞总数**<br>**Total cells / Vial**	对复苏后的细胞采取台盼蓝染色计数法<br>Cell count using Trypan Blue stain method	≥1×10^6 cells/vial	2.61×10^6
**种属鉴定**<br>**Species determination**	提取全基因组 DNA，用种属特异性引物进行 PCR 扩增<br>Involves extracting whole genome DNA and performing PCR amplification using species specific primers	人源<br>Human	人源<br>Human
**细菌/真菌**<br>**Bacteria/ Fungi**	依据 2020 年版药典无菌检查法，采取细菌/真菌直接培养法<br>According to Chinese Pharmacopoeia (2020), direct cultivation method for bacteria and fungi was used.	阴性<br>Negative	阴性<br>Negative
**支原体**<br>**Mycoplasma**	PCR 法<br>PCR method	阴性<br>Negative	阴性<br>Negative

检验结论 Conclusion： **各项指标检测通过，该批次产品检测合格。**
All specifications have been met. This batch of product has been confirmed to be qualified.

检测人/日期 Prepared by / Date： 何玉婷 2024.05.23

审核人/日期 Reviewed by / Date： [illegible] 2024.05.23

批准人/日期 Approved by / Date： [illegible] 2024.05.23

苏州海星生物科技有限公司 检测专用章

1　细胞状态 Cell state

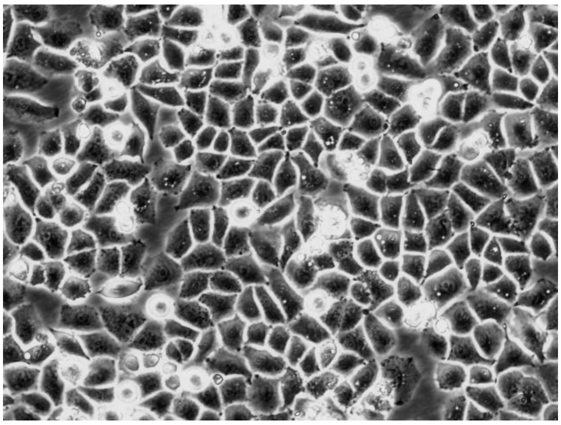

IOSE-80-240103M201-复苏 T25 后 24h-100X

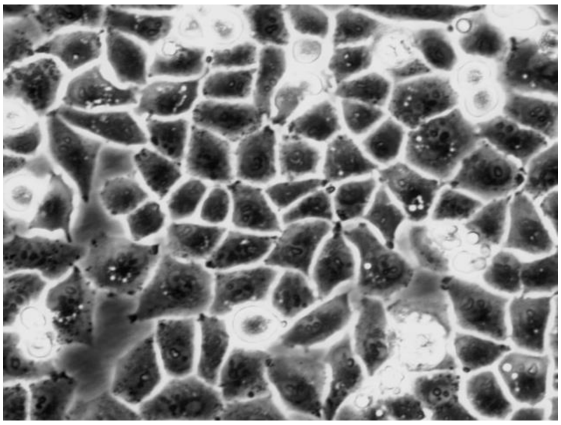

IOSE-80-240103M201-复苏 T25 后 24h-200X

2　支原体检测（PCR 法）Mycoplasma detection (PCR method)

检测结论 Test conclusion

1) 实验中阴性及阳性对照结果均成立。
2) 样品基因组 DNA 扩增后无阳性条带。
3) 细胞株中**<u>未发现支原体污染</u>**。

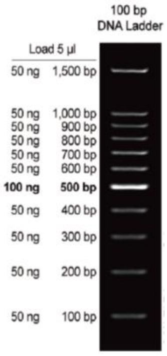

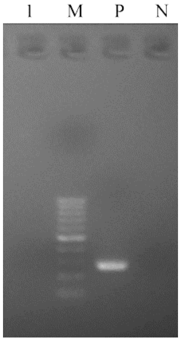

备注：泳道 1 为样品 IOSE-80（sample 1）；
泳道 M 为 Marker；泳道 P 为阳性对照；泳道 N 为阴性对照。

3　种属鉴定（PCR 法）Species identification (PCR method)

检测结论 Test conclusion

1) 实验中阳性对照结果均成立。
2) 样品基因组 DNA 扩增电泳条带清晰，分析结果良好。
3) 细胞株中**<u>未发现人、小鼠和大鼠来源交叉污染</u>**。
4) 该细胞为**<u>人源细胞</u>**。

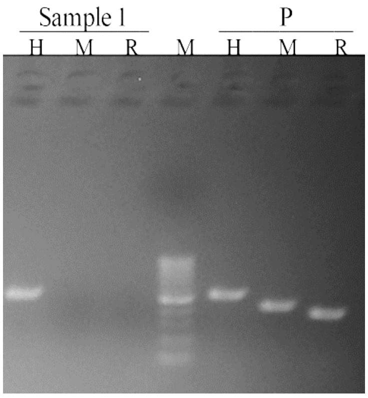

备注：第一、二、三泳道分别代表样品 IOSE-80（sample 1）分别用人、小鼠、大鼠引物检测的结果；泳道 M 为 Marker；
第五、六、七泳道分别为人、小鼠、大鼠的阳性对照。

4 Short Tandem Repeat（STR）Report

**Sample Information**	**Sample: IOSE-80**<br>**Cat. No. TCH-C402**<br>**Lot. No. 240103M201**
**Data Interpretation**	Human Cell Line Authentication Standardization of Short Tandem Repeat (STR) Profiling ASN-0002 Revised version 2021<br>The Pharmacopoeia of the People's Republic of China, Volume III General Requirements, Requirements for Preparation and Control of Animal Cell Substrates Used for Production of Biologics
**Methodology**	Sample DNA was extracted with genome extraction kit (Axygen) and PCR amplified with STR Multi-amplification Kit (MicroreaderTM21 ID System). PCR products were assayed with ABI 3730xl DNA Analyzer (Applied Biosystems®). Appropriate positive and negative controls were run and confirmed for each sample submitted. Data of 20 STR loci plus the Amelogenin locus were analyzed and extracted using Gene Mapper ID-X software and designated as the STR profile of the test sample.
**Identify Result**	The results of the negative and positive control match expectations.<br>The STR profiles of the test sample are clear, which are shown in the attached table and figure.<br>Test results for Multiple Alleles: Number of multiple alleles: 0, Locus: None.
**Explanation of Test Results**	The submitted sample profile is human, but not a match for any profile in the ExPASy STR database.

Results of database comparison (the highest percent match to the query)

Loci	Test Results for Submitted Sample			Reference Database Profile		
	Sample			**Database Profile：IOSE-80**		
	Allele1	Allele2	Allele3	Allele1	Allele2	Allele3
**D5S818**	11	12		11	12	
**D13S317**	14	14		14	14	
**D7S820**	10	11		10	11	
**D16S539**	11	14		11	14	
**VWA**	16	16		16	17	
**TH01**	6	6		6	6	
**AMEL**	X	X		X	X	
**TPOX**	8	10		8	10	
**CSF1PO**	11	12		11	12	
**D12S391**	20	22				
**FGA**	20	24				
**D2S1338**	21	22				
**D21S11**	28	29				
**D18S51**	16	16				
**D8S1179**	15	15				
**D3S1358**	14	16				
**D6S1043**	10	19				
**PENTAE**	9	13				
**D19S433**	12	12				
**PENTAD**	8	9				
**D1S1656**	12	13				

Figure 1: Genotyping of STR and Amelogenin loci in cells

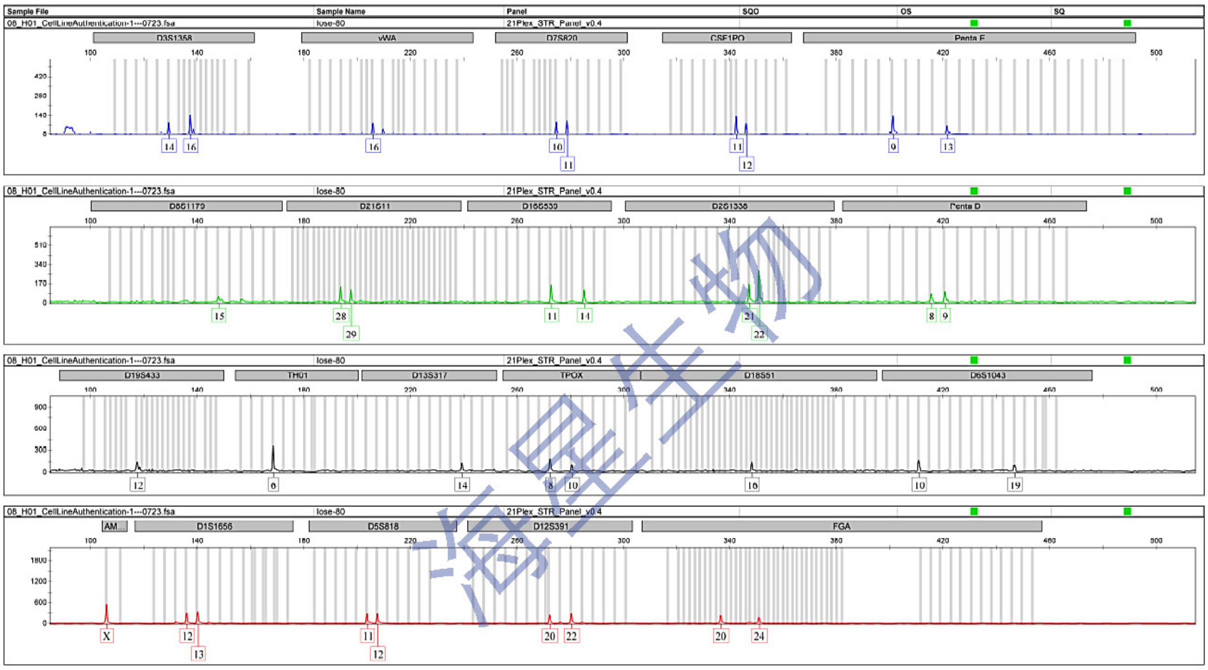

海星生物

***报告结束 END OF REPORT ***
