# Supplementary material for: A Multiomics and Bioinformatics Analysis of the Role and Mechanism of CNKSR1 in Ovarian Cancer Progression
Source: Int J Genomics. 2026 Apr 11;2026:1546020. doi: 10.1155/ijog/1546020 (PMC13069470; doi:10.1155/ijog/1546020)
Supplement: Supplementary file 1 — Supporting Information Additional supporting information can be found online in the Supporting Information section. [file IJOG-2026-1546020-s001.zip › 细胞鉴定图.zip!/τ╗åΦâ₧Θë┤σ«Üσ¢╛/HEY_Σ║║σì╡σ╖óτÖîτ╗åΦâ₧_TCH-C449_240418N201_A5.pdf]

成品检验报告书
Certificate of Analysis

产品名称 Product Name: **人卵巢癌细胞（HEY）**

产品货号 Cat. No. **TCH-C449**

产品批号 Lot. No. **240418N201**

检验依据 Standards: **现行版《中国药典》三部和企业内控标准**
The current version of the Chinese Pharmacopoeia & enterprise internal control standards.

检测项目<br>Content	检测方法<br>Method	检测标准<br>Specification	检测结果<br>Results
**生长特性**<br>**Growth properties**	常规培养<br>Conventional cultivation	贴壁细胞<br>Adherent	贴壁细胞<br>Adherent
**细胞活率**<br>**Post-freeze viability**	对复苏后的细胞采取台盼蓝染色计数法<br>Cell count using Trypan Blue stain method	≥80%	94.18%
**细胞总数**<br>**Total cells / Vial**	对复苏后的细胞采取台盼蓝染色计数法<br>Cell count using Trypan Blue stain method	≥1×10^6 cells/vial	1.07×10^6
**种属鉴定**<br>**Species determination**	提取全基因组 DNA，用种属特异性引物进行 PCR 扩增<br>Involves extracting whole genome DNA and performing PCR amplification using species specific primers	人源<br>Human	人源<br>Human
**STR 鉴定**<br>**STR identification**	根据 STR 分型结果与数据库进行比对<br>Compare the STR typing results with the database	匹配率≥0.8<br>Matching≥0.8	0.97
**细菌/真菌**<br>**Bacteria/ Fungi**	依据 2020 年版药典无菌检查法，采取细菌/真菌直接培养法<br>According to Chinese Pharmacopoeia (2020), direct cultivation method for bacteria and fungi was used.	阴性<br>Negative	阴性<br>Negative
**支原体**<br>**Mycoplasma**	PCR 法<br>PCR method	阴性<br>Negative	阴性<br>Negative

检验结论 Conclusion： **各项指标检测通过，该批次产品检测合格。**
All specifications have been met. This batch of product has been confirmed to be qualified.

检测人/日期 Prepared by / Date： 何玉婷 2024.06.15

审核人/日期 Reviewed by / Date： [illegible] 2024.06.15

批准人/日期 Approved by / Date： [illegible] 2024.06.15

苏州海星生物科技有限公司 检测专用章

1　细胞状态 Cell state

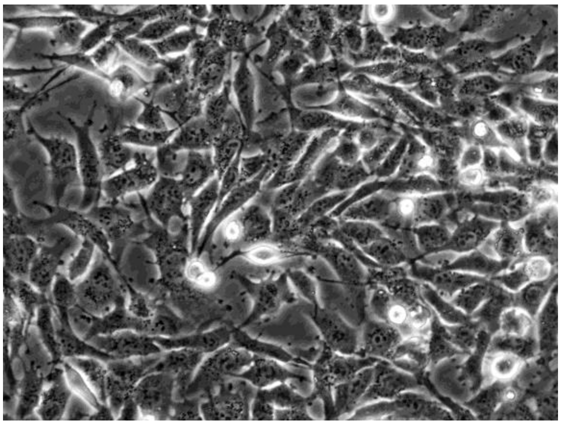

HEY-240418N201-复苏 T25 后 24h-100X

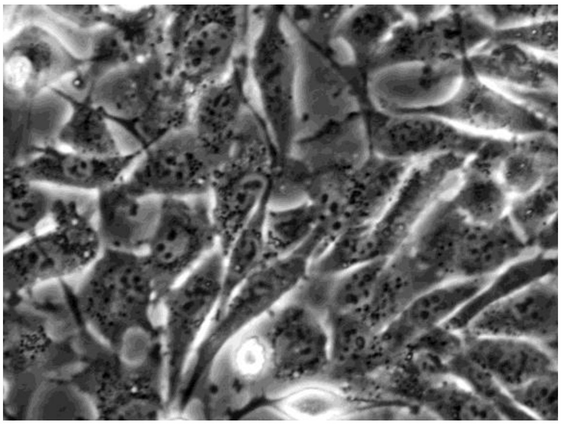

HEY-240418N201-复苏 T25 后 24h-200X

2　支原体检测（PCR 法）Mycoplasma detection (PCR method)

检测结论 Test conclusion

1)　实验中阴性及阳性对照结果均成立。

2)　样品基因组 DNA 扩增后无阳性条带。

3)　细胞株中**未发现支原体污染**。

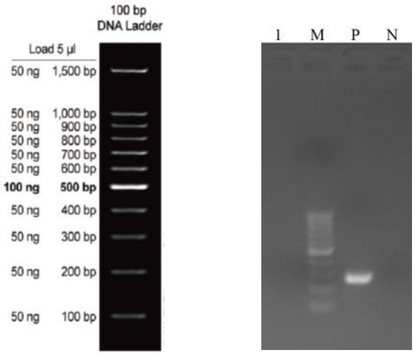

备注：泳道 1 为样品 HEY（sample 1）；
泳道 M 为 Marker；泳道 P 为阳性对照；泳道 N 为阴性对照。

3　种属鉴定（PCR 法）Species identification (PCR method)

检测结论 Test conclusion

1)　实验中阳性对照结果均成立。

2)　样品基因组 DNA 扩增电泳条带清晰，分析结果良好。

3)　细胞株中**未发现人、小鼠和大鼠来源交叉污染**。

4)　该细胞为**人源细胞**。

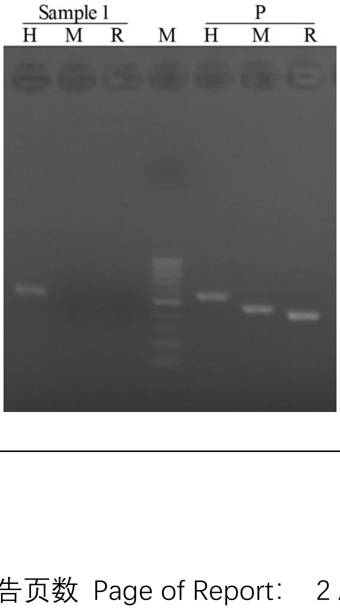

备注：第一、二、三泳道分别代表样品 HEY（sample 1）分别用人、小鼠、大鼠引物检测的结果；泳道 M 为 Marker；
第五、六、七泳道分别为人、小鼠、大鼠的阳性对照。

4 Short Tandem Repeat（STR）Report

**Sample Information**	**Sample: HEY**<br>**Cat. No. TCH-C449**<br>**Lot. No. 240418N201**
**Data Interpretation**	Human Cell Line Authentication Standardization of Short Tandem Repeat (STR) Profiling ASN-0002 Revised version 2021<br>The Pharmacopoeia of the People's Republic of China, Volume III General Requirements, Requirements for Preparation and Control of Animal Cell Substrates Used for Production of Biologics
**Methodology**	Sample DNA was extracted with genome extraction kit (Axygen) and PCR amplified with STR Multi-amplification Kit (MicroreaderTM21 ID System). PCR products were assayed with ABI 3730xl DNA Analyzer (Applied Biosystems®). Appropriate positive and negative controls were run and confirmed for each sample submitted. Data of 20 STR loci plus the Amelogenin locus were analyzed and extracted using Gene Mapper ID-X software and designated as the STR profile of the test sample.
**Identify Result**	The results of the negative and positive control match expectations.<br>The STR profiles of the test sample are clear, which are shown in the attached table and figure.<br>Test results for Multiple Alleles: Number of multiple alleles: 0, Locus: None.
**Explanation of Test Results**	The submitted sample profile is human.<br>The submitted profile has a 97% match on 9 loci of the following human cell line(s) in Expasy database：HEY.

Results of database comparison (the highest percent match to the query)

Loci	Test Results for Submitted Sample			Reference Database Profile		
	Sample			**Database Profile：HEY**		
	Allele1	Allele2	Allele3	Allele1	Allele2	Allele3
**D5S818**	11	12		11	12	13
**D13S317**	11	11		11	11	
**D7S820**	12	12		12	12	
**D16S539**	8	12		8	12	
**VWA**	16	17		16	17	
**TH01**	8	9.3		8	9.3	
**AMEL**	X	X		X	X	
**TPOX**	11	11		11	11	
**CSF1PO**	10	11		10	11	
**D12S391**	17	22				
**FGA**	20	21				
**D2S1338**	24	25				
**D21S11**	30	30				
**D18S51**	15	15				
**D8S1179**	13	13				
**D3S1358**	16	16				
**D6S1043**	11	12				
**PENTAE**	7	13				
**D19S433**	13	14				
**PENTAD**	9	13				
**D1S1656**	12	15				

Figure 1: Genotyping of STR and Amelogenin loci in cells

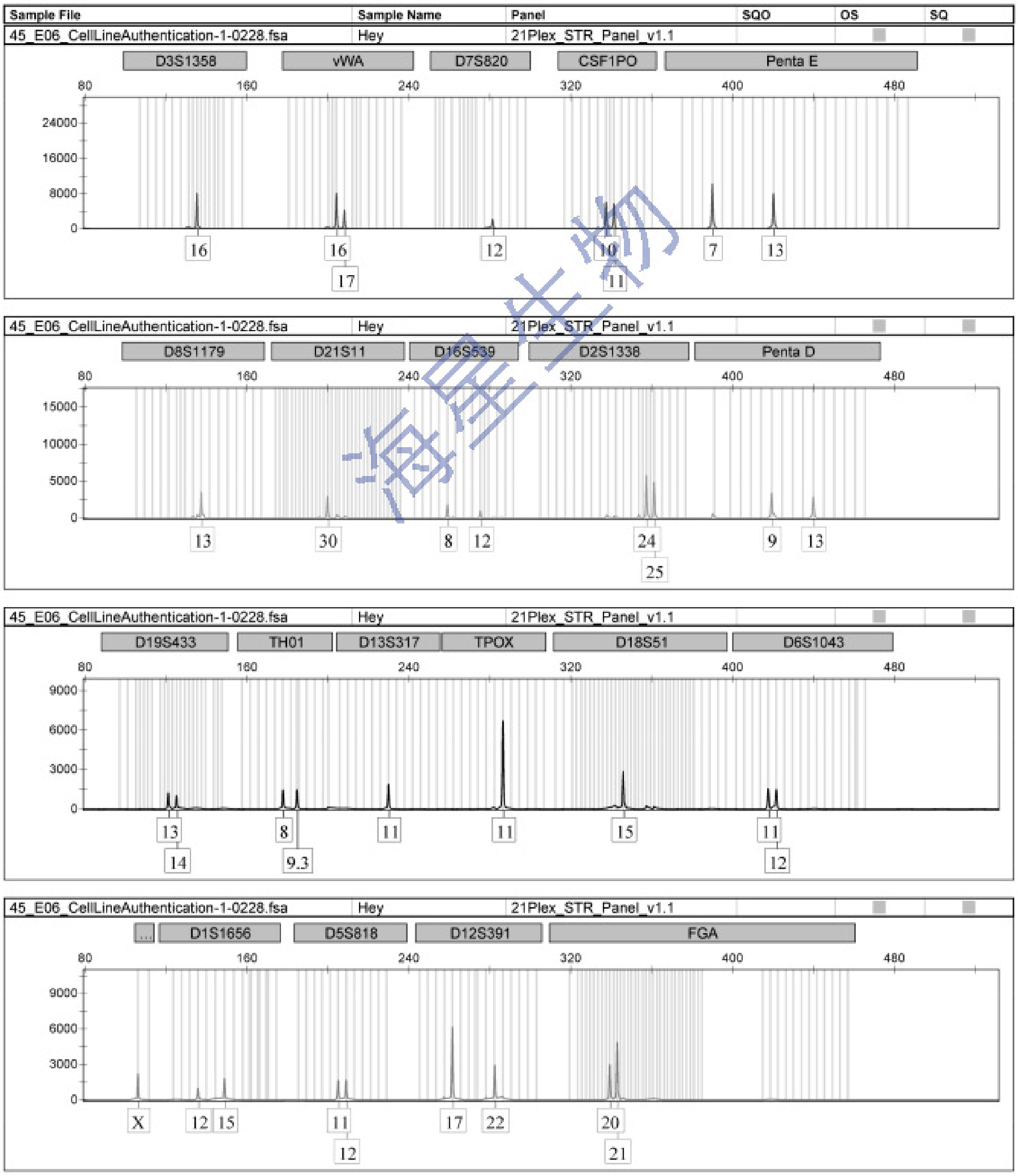

海星生物

***报 告 结 束　END OF REPORT ***
